# Lethal Phenotype-Based Database Screening Identifies Ceramide as a Negative Regulator of Primitive Streak Formation

**DOI:** 10.1093/stmcls/sxad071

**Published:** 2023-10-11

**Authors:** Jing Pu, Satoshi Kofuji, Yoshimi Okamoto-Uchida, Keiko Danzaki, Ruoxing Yu, Akira Suzuki, Satoshi Kitajima, Hiroshi Nishina

**Affiliations:** Department of Developmental and Regenerative Biology, Medical Research Institute, Tokyo Medical and Dental University, Tokyo, Japan; Department of Developmental and Regenerative Biology, Medical Research Institute, Tokyo Medical and Dental University, Tokyo, Japan; Department of Developmental and Regenerative Biology, Medical Research Institute, Tokyo Medical and Dental University, Tokyo, Japan; Department of Developmental and Regenerative Biology, Medical Research Institute, Tokyo Medical and Dental University, Tokyo, Japan; Department of Developmental and Regenerative Biology, Medical Research Institute, Tokyo Medical and Dental University, Tokyo, Japan; Division of Molecular and Cellular Biology, Kobe University Graduate School of Medicine, Kobe, Japan; Division of Cellular and Molecular Toxicology, Center for Biological Safety and Research, National Institute of Health Sciences, Kawasaki, Japan; Department of Developmental and Regenerative Biology, Medical Research Institute, Tokyo Medical and Dental University, Tokyo, Japan

**Keywords:** primitive streak, cardiac differentiation, neural differentiation, ceramide, sphingosine-1-phosphate

## Abstract

In early embryogenesis, the primitive streak (PrS) generates the mesendoderm and is essential for organogenesis. However, because the PrS is a minute and transient tissue, elucidating the mechanism of its formation has been challenging. We performed comprehensive screening of 2 knockout mouse databases based on the fact that failure of PrS formation is lethal. We identified 812 genes involved in various cellular functions and responses that might be linked to PrS formation, with the category of greatest abundance being “Metabolism.” In this study, we focused on genes of sphingolipid metabolism and investigated their roles in PrS formation using an in vitro mouse ES cell differentiation system. We show here that elevated intracellular ceramide negatively regulates gene expression essential for PrS formation and instead induces neurogenesis. In addition, sphingosine-1-phosphate (a ceramide derivative) positively regulates neural maturation. Our results indicate that ceramide regulates both PrS formation and the induction of neural differentiation.

## Graphical Abstract

**Graphical Abstract:**
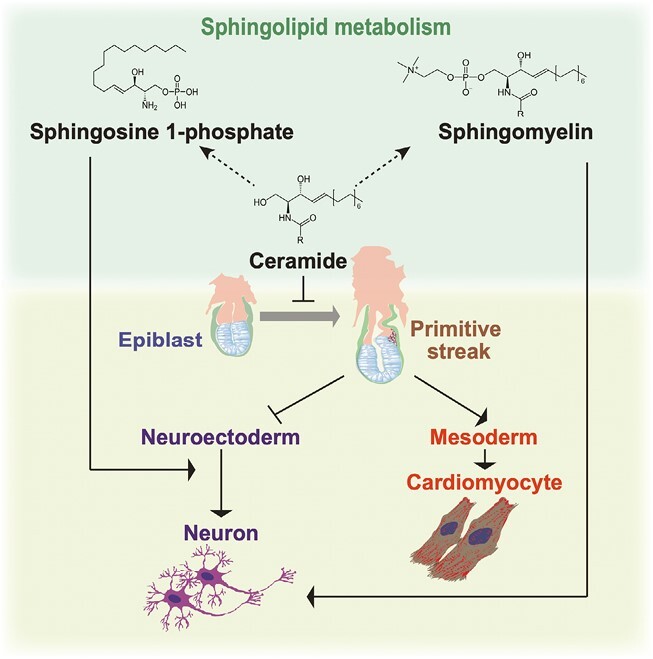

**Significance Statement**

Understanding the detailed molecular mechanisms of embryogenesis is necessary for advances not only in embryology but also in regenerative medicine. Primitive streak formation is the key first step controlling the differentiation of both the mesendoderm and ectoderm. In this study, we have identified a metabolic pathway crucial for regulating primitive streak formation. Our findings thus reveal a critical mechanism controlling how distinct cell lineages differentiate, information that may prove valuable in regenerative medicine approaches based on embryonic stem cell manipulation.

## Introduction

During early embryogenesis, individual cells receive various signals and influence each other to trigger complex events. Elucidating the regulatory mechanisms involved in these events should lead to advances not only in developmental biology but also in related fields such as regenerative medicine. In early vertebrate embryos, all organs are formed from 3 germ layers: the ectoderm, mesoderm, and endoderm.^1,2^ The mesoderm and endoderm both arise from the mesendoderm in a process requiring the epiblast to form a cellular groove called the primitive streak (PrS). However, the PrS is a minute and transient tissue, which has made it difficult to fully dissect the molecular mechanisms of its formation.

Embryonic stem (ES) cell differentiation systems are powerful in vitro tools for studying the processes driving basic development from the epiblast.^3,4^ When ES cells are cultured in suspension, they form a multicellular assembly called the embryoid body (EB), which has properties similar to those of an epiblast in vivo. In both mice^3^ and humans,^5^ EBs can be induced to differentiate into the 3 germ layers in vitro. This approach has been frequently used to differentiate mouse ES cells into various cell types, including cardiomyocytes,^6^ neurons,^7^ and hepatocytes,^8^ and has permitted analyses of the underlying mechanisms. Recently, the entire process of oogenesis from mouse ES cells was reconstituted in vitro,^9^ and these in vitro-generated eggs successfully gave rise to a birth. In humans, structures resembling blastocysts in terms of their morphology, size, cell number, and cell lineage composition have been generated from human ES cells in vitro.^10^

We previously identified several factors essential for PrS formation by screening a library of 1600 chemical compounds with known targets.^11^ In our experimental system, mouse ES cells undergo EB formation, followed by passage of the cells through an epiblast-like state. PrS formation is then mimicked, complete with expression of PrS markers such as *Brachyury T* and *Wnt3* on day 3 to day 4. Cell beating (differentiation into cardiomyocytes) is observed on day 10 after EB formation, accompanied by expression of mesoderm markers such as *Bmp2*. We used this EB-based system to show that various metabolic inhibitors block PrS formation, and demonstrated that chemical screening is useful for identification of factors critical for PrS formation.^12,13^ Interestingly, in all of these cases, when PrS formation was inhibited, the epiblast cells committed to the neuroectodermal lineage, and cardiomyocyte differentiation from mesendoderm was decreased. These results indicated that PrS formation influences neural differentiation, an observation that inspired the current study.

Although screening a library of drug compounds with known targets has the advantage of immediate identification of the relevant genes, it has the disadvantage that some enzymes or receptors may not correspond to drugs in the library, preventing their validation. To circumvent this difficulty, we exploited the fact that knockout (KO) mice deficient for the function of genes such as *Brachyury T* and *HMGCR*, which are essential for PrS formation, are lethal by E10 due to defects in the development of organs such as the heart.^14,15^ By exploiting this lethal phenotype, we aimed to examine a wider range of genes and obtain a more complete picture of the mechanisms underlying PrS formation. To this end, we screened 2 publicly available KO mouse databases to identify genes whose loss of function causes E10 lethality in mouse embryos. We then used our in vitro mouse ES cell differentiation system to identify ceramide as a novel factor involved in PrS formation.

## Materials and Methods

### Database Screening

Candidate genes were selected by screening the MGI database (Mouse Genome Informatics, http://www.informatics.jax.org/) and the IMPC database (International Mouse Phenotyping Consortium)^16,17^ as described in the Results. The data used in this study were current as of May 2021. In the MGI database, the phenotype term “embryonic lethality between implantation and placentation” (ID: MP:0009850) means that embryonic death occurred between E4.5 and E9; this criterion was used for selecting candidate genes. The term “embryonic lethality, complete penetrance,” which means embryonic death occurred before E14, was used as a supplemental criterion. The IMPC database annotates KO mouse lines and reports their lethality phenotype. Genes for which the corresponding KO mice died before E9 were selected as candidates for our study. For the network analysis, NetworkAnalysist 3.0 (https://www.networkanalyst.ca) was used.^18^

### ES Cell Culture and Differentiation

129P2/OlaHsd-derived E14K embryonic stem (mES) cells (kindly provided by Tak W. Mak of the Princess Margaret Cancer Centre, Toronto) were maintained in an undifferentiated state by culture in the presence of leukemia inhibitory factor (LIF) as described previously.^19-21^ Briefly, feeder cell-independent E14K mES cells were maintained in gelatin-coated dishes containing Dulbecco’s modified Eagle’s medium (26400044; Gibco, MA, USA) supplemented with 15% bovine calf serum (SFBM30-2362; Equitech-bio, TX, USA), 0.1 mM 2-mercaptoethanol (M3148; Sigma, Burlington, USA) and LIF.

mES cells (3 × 10^3^) were cultured in 25 µL hanging drops in medium without LIF and allowed to form EBs in a square dish (Eiken Chemical, Tokyo, Japan). After 2 days, EBs were transferred into a non-coated bacterial Petri dish (IWAKI, Tokyo, Japan) for suspension culture. On day 6, EBs were transferred into a gelatin-coated tissue culture dish (Corning, NY, USA) for attachment culture until day 10, when the areas of tissue showing a spontaneous “heartbeat” can be detected by microscopy. The “beating ratio” was calculated as the percentage of beating EBs among all EBs observed, and the “neurite ratio” was the percentage of EBs with β-tubulin III-positive protrusions among all EBs observed. EBs were treated with inhibitor drugs during days 3-6 of culture unless otherwise noted.

### Immunofluorescence Staining

Immunofluorescence staining was performed as described previously.^11,12,22,23^ Briefly, EBs were fixed for 2 h or overnight in 4% paraformaldehyde (PFA) in phosphate-buffered saline (PBS), followed by 3 washes in PBS. After preincubation with blocking solution (1% BSA, 0.1% Triton X-100 in PBS) for 30 minutes, cells were incubated overnight at 4 °C with a 1:1000 dilution of anti-β-tubulin III antibody. After 3 washes in PBS/0.1% Triton X-100, EBs were incubated with a 1:1000 dilution of Alexa Fluor 546-conjugated anti-mouse antibody. Stained EBs were washed 3 times in PBS/0.1% Triton X-100 before examination using a fluorescence microscope BZ-X710 (Keyence, Osaka, Japan).

### Statistical Analyses

Analyses of statistical significance were performed using GraphPad Prism 8 (GraphPad Software Inc., San Diego, CA). Student’s t test was used for comparisons between 2 groups. One-way analysis of variance (ANOVA) was used for comparisons among 3 or more groups. Two-way ANOVA was used for 2-factor analysis. Data were considered statistically significant at *P* < .05. Principal component analysis (PCA) was performed using MetaboAnalyst 5.0.

Other detailed methods are provided in the Supplementary Methods and include the following:

Reagents and antibodies
Whole mount in situ hybridization
Metabolomic analysis
RNA sequencing and analysis
Real-time PCR analysis
Lipid extraction and phosphatidylcholine measurement
Ceramide measurement

## Results

### Lethality-Based Screen Identifies Genes Essential for Organogenesis

**Figure 1.**
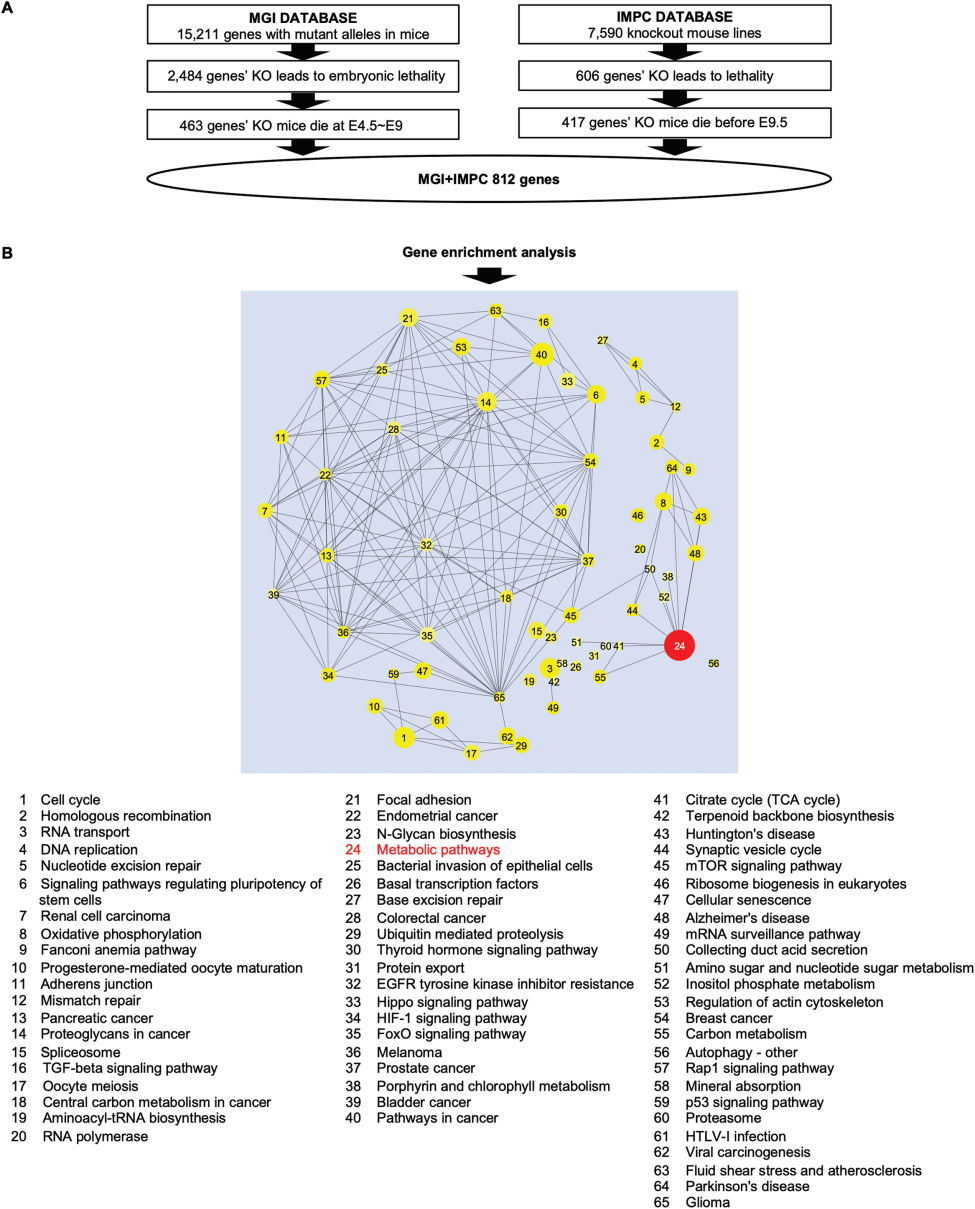
Comprehensive screening of knockout mouse databases for genes whose loss induces embryonic lethality by E10. (**A**) Diagram of the scheme used for KO mouse database screening. See main text for details. (**B**) Enrichment analysis. The circle size reflects the number of genes assigned to each term. The largest group, “Metabolic pathways,” is highlighted in red.

To comprehensively identify genes involved in PrS formation, we screened 2 KO mouse databases, the MGI database (15 211 genes) and the IMPC database (7590 KO mouse lines), and looked for genes whose loss led to embryonic lethality by E10 (Fig. 1A). Of genes in the MGI database, loss of any one of 463 genes was found to be embryonic lethal between E4.5 and E9 (Supplementary Table S1). Similarly, of genes in the IMPC database, loss of any one of 417 genes resulted in embryonic lethality before E9.5 (Supplementary Table S2). Together, a total of 812 genes of interest was identified, including, as expected, the PrS-regulated gene *Brachyury T* (Supplementary Table S3).

Enrichment analysis of the 812 candidate genes identified 65 cellular functional pathways of interest (Fig. 1B; Supplementary Table S4). These functional pathways formed a network involving genes needed for DNA replication, RNA metabolism, energy metabolism, and signal transduction, as well as diverse cellular responses such as the cell cycle and cell adhesion. The category encompassing the highest number (103) of genes of interest was “Metabolic pathways” (No. 24) (Fig. 1B and Supplementary Table S4). We then classified these 103 genes using the Database for Annotation, Visualization and Integrated Discovery (DAVID) and identified genes involved in glucose, mitochondrial, nucleic acid, and lipid metabolism (Supplementary Table S5 and Figs. S1-S8). These metabolic pathways are not only necessary for energy supply but are also important for synthesizing cellular components and for epigenetic regulation of gene expression, processes that are essential for proper biological activities. These results indicated that early embryonic development requires a variety of intact cellular functions, particularly metabolism.

### Inhibition of the Sphingolipid Metabolic Pathway Inhibits Cardiac Differentiation and Promotes Neural Differentiation of Mouse ES Cells

**Figure 2.**
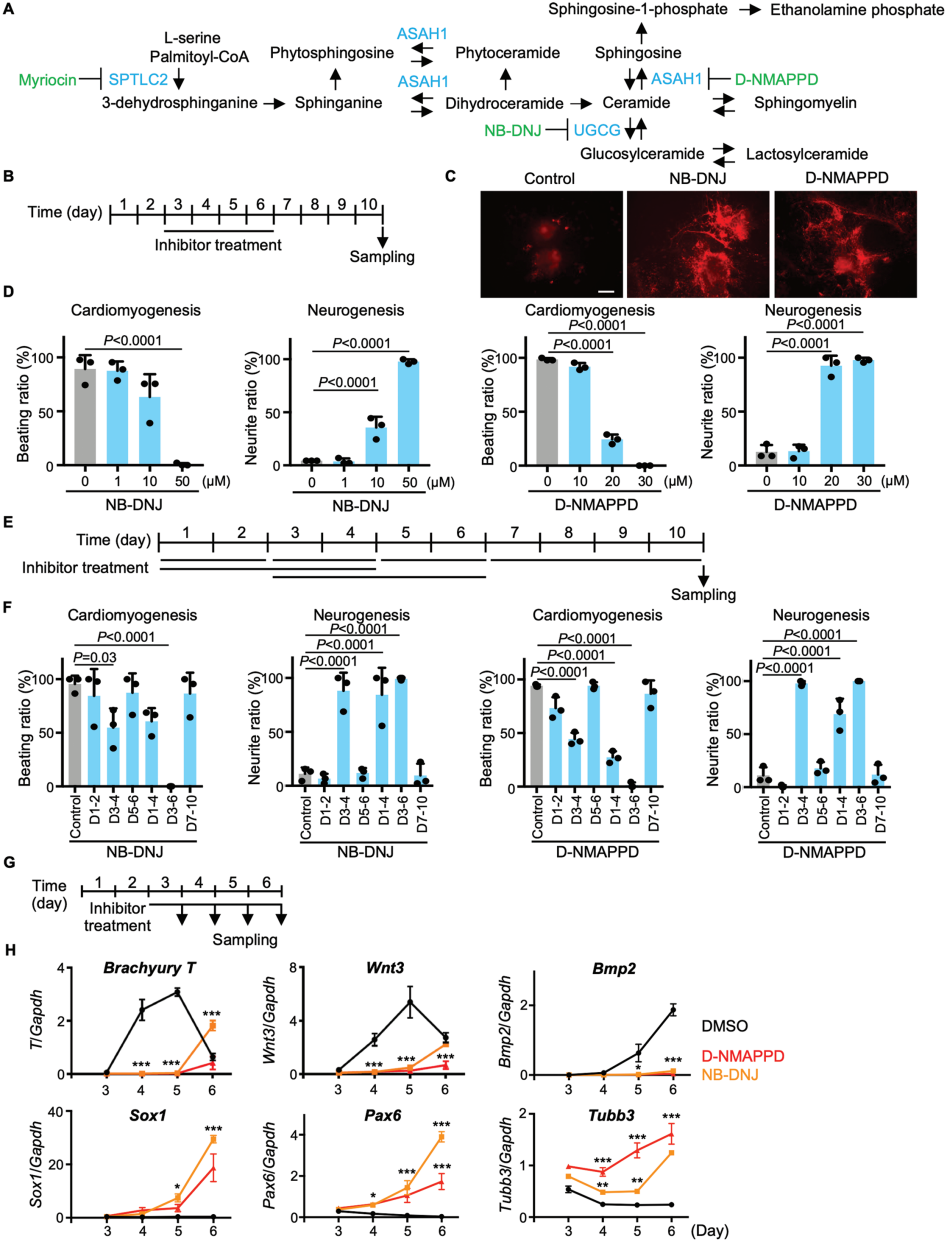
Effects of NB-DNJ and D-NMAPPD on PrS formation. (**A**) Diagram of the sphingolipid metabolic pathway. Genes encoding relevant enzymes and their specific inhibitors are shown in blue and green, respectively. (**B**) Experimental scheme for the EB differentiation assays in C and D. (**C**) Immunostaining of EBs with anti-β-tubulin III antibody to detect morphological changes after treatment with the indicated inhibitors for days 3-6. Scale bar = 400 μm. Data are representative of 3 biologically independent experiments. (**D**) Quantitation of dose-dependent effects of NB-DNJ or D-NMAPPD on EB cardiomyogenesis and neurogenesis. Data are the mean + s.d. of *n* = 3 biologically independent samples. Statistical analysis was performed by one-way ANOVA with the Tukey test. (**E**) Experimental scheme for the time course experiments in F. (**F**) Quantitation of cardiomyogenesis and neurogenesis in EBs that were treated with 50 μM NB-DNJ or 30 μM D-NMAPPD for the indicated periods. Data are the mean + s.d. of *n* = 3 biologically independent samples. Statistical analysis was performed by one-way ANOVA with the Tukey test. (**G**) Experimental scheme for the time course experiments in H. (**H**) Real-time PCR analysis of indicated genes’ mRNA levels in EBs treated with DMSO, 50 μM NB-DNJ, or 50 μM D-NMAPPD for days 3-6. Data are expressed relative to *Gapdh* and are the mean ± s.d. of *n* = 3 biologically independent samples. Statistical analysis was performed by 2-way ANOVA with the Dunnett’s multiple comparison test. *P < .05, **P < .01, ***P < .001.

We previously observed increases in sphingomyelin and sphingosine in EBs upon failure of PrS formation caused by inhibition of mevalonate metabolism.^11^ To determine the biological significance of these changes, we focused on sphingolipid metabolism in the present study. Three genes (Serine Palmitoyltransferase Long Chain Base Subunit 2 (*Sptlc2*), UDP-Glucose Ceramide Glucosyltransferase (*Ugcg*), and N-Acylsphingosine Amidohydrolase 1 (*Asah1*)) are known to contribute to sphingolipid metabolism (Fig. 2A). To investigate the roles of the SPTLC2, UGCG, and ASAH1 proteins in PrS formation, we examined the effects of specific inhibitors of these enzymes (Myriocin^24,25^ for SPTLC2, NB-DNJ^26,27^ for UGCG, and D-NMAPPD^28-31^ for ASAH1) on mouse ES cell differentiation (Fig. 2A). Inhibitors were applied for days 3-6 of culture, and ES cell differentiation was analyzed on day 10 by light microscopy to detect cardiomyocyte beating, and by β-tubulin III immunostaining to detect neurite formation (Fig. 2B). We found that Myriocin inhibited both cardiomyocyte beating and the formation of β-tubulin III-positive neurites, resulting in cell death before PrS formation (Supplementary Fig. S9). In contrast, while both NB-DNJ and D-NMAPPD inhibited EB beating, these agents also promoted the formation of β-tubulin III-positive neurites in a dose-dependent manner (Fig. 2C, D). These data implied that UGCG and ASAH1 are important for normal PrS formation.

To elucidate at which time point inhibition of UGCG by NB-DNJ or blockage of ASAH1 by D-NMAPPD impeded cell beating, we treated cells with each inhibitor for 6 time periods (days 1-2, 3-4, 5-6, 7-10, 1-4, and 3-6) and analyzed cell beating and neurite outgrowth on day 10 (Fig. 2E). NB-DNJ treatment on days 1-4, 3-4, and 3-6 efficiently depressed cell beating and promoted neurite outgrowth (Fig. 2F, left). Similar results were observed when ASAH1 was inhibited using D-NMAPPD (Fig. 2F, right). Thus, days 3-6 of EB development appear to be particularly dependent on sphingolipid metabolism.

To define the spatio-temporal effects of UGCG inhibition on PrS formation, we performed in situ hybridization to detect expression of the PrS marker *Brachyury T*. EBs were treated with NB-DNJ for days 3-4 or 3-6, and *Brachyury T* levels were examined on days 3, 4, 5 and 6 (Supplementary Fig. S10A). In control untreated EBs, *Brachyury T* expression was not seen on day 3 but peaked on day 4, gradually decreasing from day 5 to day 6 (Supplementary Fig. S10B). On the other hand, NB-DNJ treatment from days 3-6 largely suppressed *Brachyury T* expression. Interestingly, when NB-DNJ was applied only from days 3-4, *Brachyury T* expression was suppressed on day 4 but had recovered by day 5 following the removal of NB-DNJ. Real-time PCR experiments confirmed that NB-DNJ and D-NMAPPD treatment from days 3-6 attenuated the induction of PrS markers such as *Brachyury T*, *Wnt3*, and *Bmp2* (Fig. 2G and 2H, top). Instead, the drug treatment induced mRNA expression of neural markers such as *Sox1*, *Pax6*, and *Tubb3* (Fig. 2H, bottom). Thus, inhibition of ASAH1 or UGCG at days 3-4 of EB development blocks PrS formation and inhibits cardiomyocyte differentiation while inducing neural differentiation.

### UGCG Inhibition in EBs Decreases Lactosylceramide While Increasing Sphingomyelin

**Figure 3.**
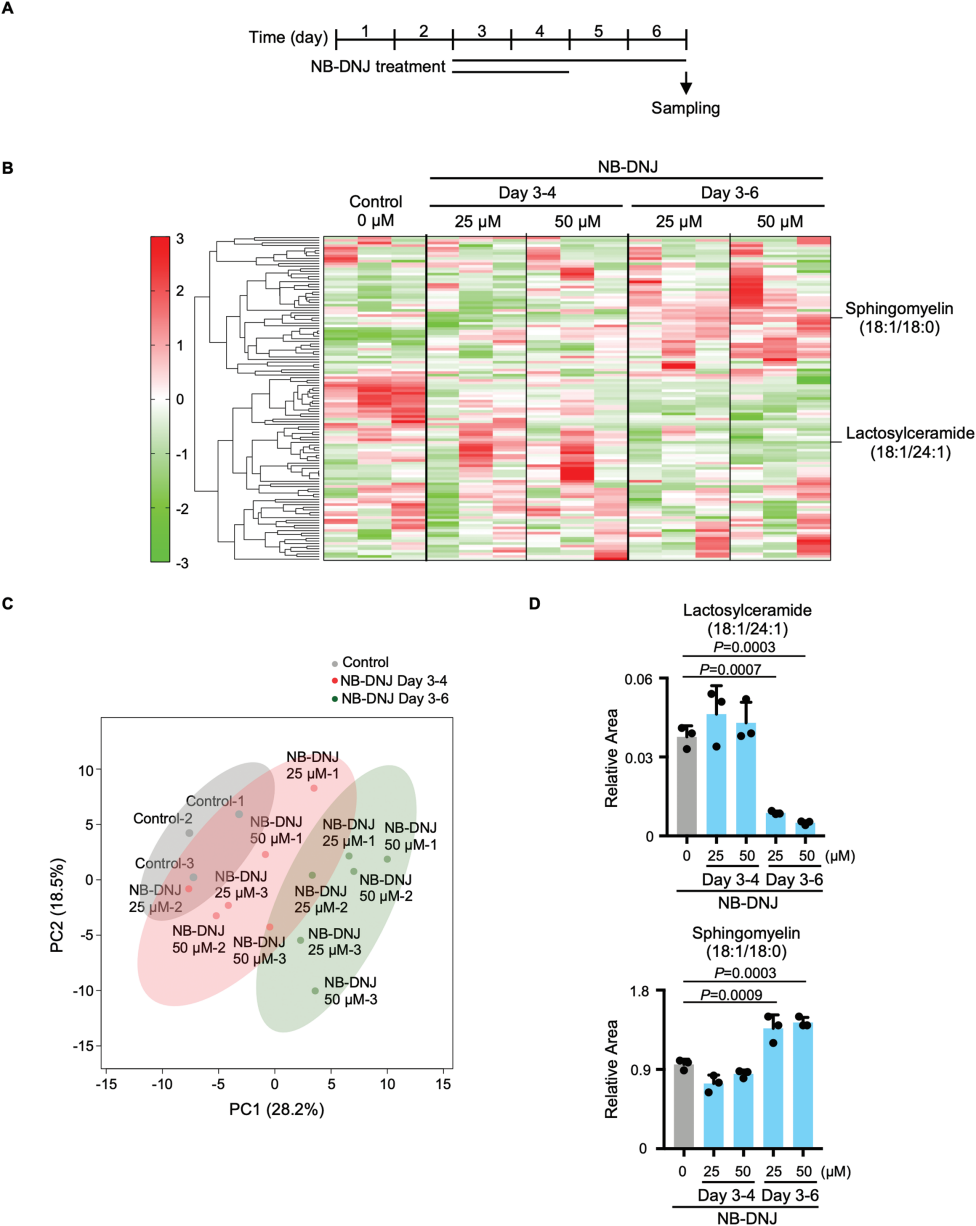
Effects of NB-DNJ on lipid metabolism. (**A**) Experimental scheme for metabolomic analysis of NB-DNJ-treated EBs. (**B**) Clustering analysis of lipid metabolites in control EBs and in EBs treated with the indicated concentrations of NB-DNJ for the indicated periods. Red, upregulated metabolites; green, downregulated metabolites. (**C**) PCA plot of lipid metabolites in the control and NB-DNJ-treated EBs in B. (**D**) Quantitation of lactosylceramide (upper panel) and sphingomyelin (lower panel) in EBs treated with NB-DNJ as indicated. Data are expressed as the relative area, which indicates the amount of a metabolite normalized to internal standards, and are the mean + s.d. of *n* = 3 biologically independent samples. Statistical analysis was performed by one-way ANOVA with the Tukey test.

To investigate the changes in sphingolipid metabolism induced by UGCG inhibition, we analyzed lipophilic metabolites using LC-TOF/MS. EBs were treated with NB-DNJ on days 3-4 or 3-6 of development and metabolites were extracted on day 6 (Fig. 3A), leading to the identification of 124 peaks representing lipophilic metabolites (Supplementary Table S6). Clustering analysis revealed that NB-DNJ treatment induced time-dependent changes in metabolite abundance (Fig. 3B). Principal component analysis confirmed a time-dependent change in metabolite abundance relative to the PC1 axis (Fig. 3C), with the major contributors to the PC1 axis being sphingolipid-related metabolites such as lactosylceramide and sphingomyelin (Supplementary Table S7). NB-DNJ treatment for days 3-6 (but not days 3-4) decreased lactosylceramide levels and conversely increased sphingomyelin levels (Fig. 3D). These results indicated that, as expected (Fig. 2A), ceramide accumulates in response to UGCG inhibition.

Next, we measured ceramide levels in EBs directly. To this end, samples were taken at 0, 6, and 24 hours after NB-DNJ treatment of day 3 EBs. After 24 hour of incubation with NB-DNJ, EBs had accumulated significant levels of a specific species of ceramide (C16:0) while also showing decreases in galactosyl/glucosylceramide and lactosylceramide (Supplementary Fig. S11). Thus, UGCG inhibition in EBs leads to an increase in intracellular ceramide and thus altered levels of ceramide-related metabolites such as galactosyl/glucosylceramide, lactosylceramide, and sphingomyelin.

### Intracellular Ceramide Inhibits Cardiac Differentiation and induces Neural Differentiation

**Figure 4.**
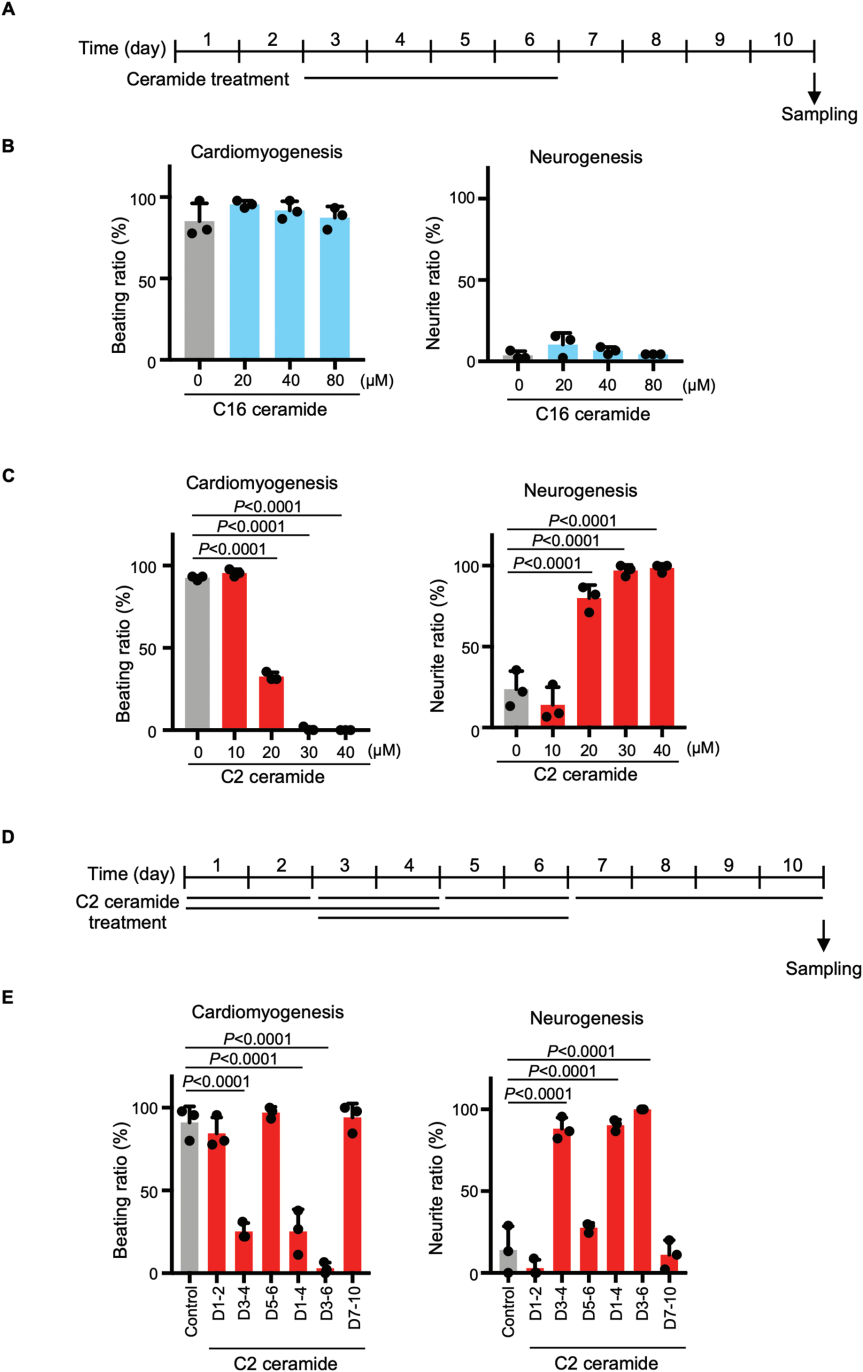
Effects of ceramides on cardiomyogenesis and neurogenesis. (**A**) Experimental scheme for the EB differentiation experiments in B and C. (**B**) Quantitation of cardiomyogenesis and neurogenesis in EBs that were treated with the indicated concentrations of C16 ceramide for days 3-6. Data are the mean + s.d. of *n* = 3 biologically independent samples. Statistical analysis was performed by one-way ANOVA with the Tukey test. (**C**) Quantitation of cardiomyogenesis and neurogenesis in EBs that were treated with the indicated concentrations of C2 ceramide for days 3-6. Data are the mean + s.d. of *n* = 3 biologically independent samples. Statistical analysis was performed by one-way ANOVA with the Tukey test. (**D**) Experimental scheme for time course experiments in E. (**E**) Quantitation of cardiomyogenesis and neurogenesis in EBs treated with 30 μM C2 ceramide for the indicated periods. Data are the mean + s.d. of *n* = 3 biologically independent samples. Statistical analysis was performed by one-way ANOVA with the Tukey test.

Ceramide functions both at the plasma membrane and as a signaling molecule within the cell.^32^ We therefore examined the effect on EBs of treatment on days 3-6 with one of 2 types of ceramide: cell-impermeable C16 ceramide or cell-permeable C2 ceramide (Fig. 4A). C16 ceramide did not decrease cell beating and did not induce neurite formation over a concentration range of 20-80 µM (Fig. 4B). In contrast, C2 ceramide inhibited cell beating in a dose-dependent manner and simultaneously triggered neurite formation (Fig. 4C). Next, ES cells were treated with C2 ceramide for days 1-2, 3-4, 1-4, 3-6, 5-6, or 7-10 to elucidate at which time point this lipid blocked cardiac differentiation (Fig. 4D). We found that C2 ceramide treatment on days 3-4 efficiently inhibited cell beating and promoted neurite formation (Fig. 4E). These data implied that ceramide was responsible for the reduction in cardiac differentiation induced by UGCG inhibition.

### Ceramide Suppresses the Expression of PrS Formation-Related Genes

**Figure 5.**
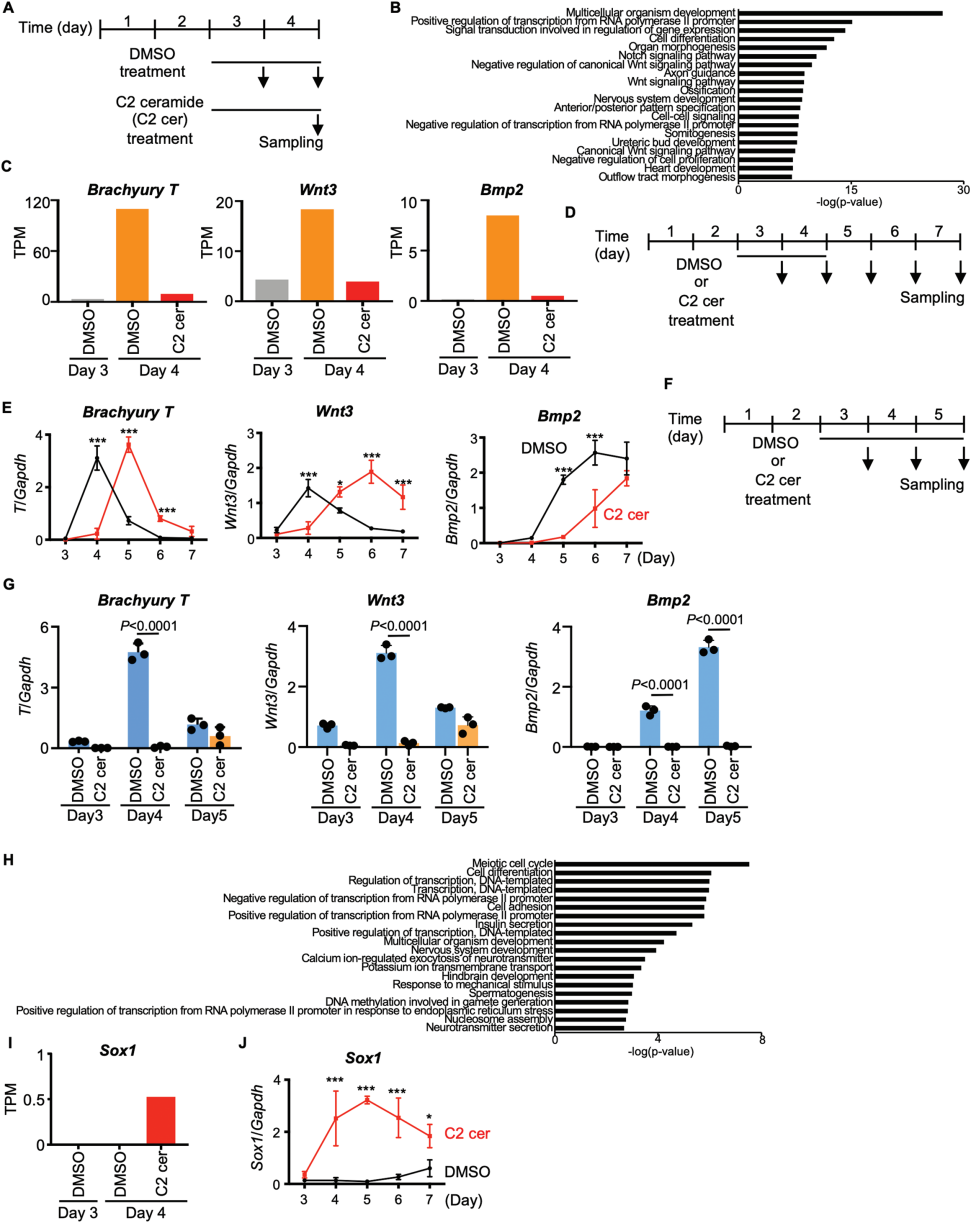
Effects of ceramide on gene expression. (**A**) Experimental scheme for the transcriptomic analysis of EBs that were treated with DMSO (vehicle control) or 30 μM C2 ceramide for days 3-4. (**B**) GO analysis of genes whose expression levels in control EBs increased on day 4 compared to day 3, but were inhibited by C2 ceramide treatment. (**C**) Quantitation of *Brachyury T*, *Wnt3* and *Bmp2* mRNA levels in the EBs in (A), as determined by RNA-sequencing analysis. Data are expressed as transcripts per million (TPM). (**D**) Experimental scheme for the time course assays in E and J. (**E**) Real-time PCR analysis of *Brachyury T*, *Wnt3* and *Bmp2* mRNA levels in EBs treated with DMSO or 30 μM C2 ceramide for days 3-4. Data are expressed relative to *Gapdh* and are the mean ± s.d. of *n* = 3 biologically independent samples. Statistical analysis was performed by 2-way ANOVA with the Bonferroni test. *P < .05, **P < .01, ***P < .001. (**F**) Experimental scheme for the real-time PCR experiments in G. (**G**) Real-time PCR analysis of *Brachyury T*, *Wnt3* and *Bmp2* mRNA levels in EBs treated with 45 μM C2 ceramide for days 3-5. Data are expressed relative to *Gapdh* and are the mean ± s.d. of *n* = 3 biologically independent samples. Statistical analysis was performed by 2-way ANOVA with the Tukey test. (**H**) GO analysis of genes whose expression levels in control EBs did not increase on day 4 compared to day 3, but were induced by C2 ceramide treatment. (**I**) Quantitation of *Sox1* mRNA levels in the EBs in (A), as determined by RNA-sequencing analysis. Data were analyzed as for (C). (**J**) Real-time PCR analysis of *Sox1* mRNA levels in EBs treated with DMSO or 30 μM C2 ceramide for days 3-4. Data were analyzed as for (E).

To examine changes in gene expression during C2 ceramide treatment, we performed transcriptomic analyses. We sought to examine 2 pools of genes: pool A, containing genes whose expression increased early during PrS formation but could be suppressed by ceramide; and pool B, containing genes that are not normally involved in early PrS formation but could be induced by ceramide. To perform these analyses, EBs were treated with solvent only (DMSO) or C2 ceramide from days 3-4, with RNA extracted on day 3 or day 4 (Fig. 5A; Supplementary Table S8).

To identify genes in pool A, we first looked for genes whose mRNA levels increased on day 4 compared to day 3 of DMSO treatment and identified 1752 such genes in DMSO-treated EBs (Supplementary Table S9 and Fig. S12A). We then measured the mRNA levels of these 1752 genes in EBs treated with C2 ceramide from days 3-4, with RNA extracted on day 3 or 4, and identified 945 genes whose expression was decreased by ceramide treatment. When these 945 genes were analyzed by Gene Ontology (GO), “Multicellular organism development” was the highest ranked term (Fig. 5B). Genes associated with “Multicellular organism development” included the PrS markers *Brachyury T* and *Wnt3*, and the mesoderm marker *Bmp2* (Fig. 5C), as well as additional genes regulating PrS formation (Supplementary Fig. S12B and S12C).

To follow the expression of *Brachyury T* and *Wnt3* over time, we treated EBs with DMSO or C2 ceramide on days 3-4, collected EBs daily on days 3-7, and analyzed extracts by real-time PCR (Fig. 5D, E). The mRNA levels of *Brachyury T* and *Wnt3* peaked on day 4 in DMSO-treated EBs (Fig. 5E), but were suppressed on day 4 in C2 ceramide-treated EBs. However, *Brachyury T* and *Wnt3* mRNA levels recovered after the cessation of C2 ceramide treatment, peaking on days 5 and 6, respectively. In contrast, expression of the mesoderm marker *Bmp2* peaked on day 6 after DMSO treatment, whereas C2 ceramide treatment suppressed *Bmp2* expression and shifted its peak to day 7 or beyond. We further examined the expression patterns of these genes in EBs continuously treated with C2 ceramide during days 3-5 (Fig. 5F) and observed almost complete suppression of *Brachyury T*, *Wnt3*, and *Bmp2* expression (Fig. 5G). These results suggested that the expression of genes essential for PrS formation can be reversibly regulated by C2 ceramide.

Next, we attempted to identify genes in pool B, which contained genes showing the opposite expression pattern; that is, genes showing no change in mRNA level on day 4 compared to day 3 of DMSO treatment of EBs, but which were increased in expression on day 4 after C2 ceramide treatment on days 3-4. We observed that, among 20 222 genes showing no change in expression in DMSO-treated EBs on day 4, the mRNA levels of 1279 of them were increased on day 4 by C2 ceramide treatment (Supplementary Table S10 and Fig. S12D). GO analysis of these 1279 genes showed that “Multicellular organism development” and “Nervous system development” were among the top 20 terms (Fig. 5H). The “Multicellular organism development” category included the neural differentiation markers *Neurod1* and *Pax6* (Supplementary Fig. S12E). The “Nervous system development” category included the neural differentiation markers *Sox1* and *Neurod1*, whose expression levels on day 4 were increased by C2 ceramide treatment (Fig. 5I and Supplementary Fig. S12E and S12F). Real-time PCR analysis of time-dependent *Sox1* expression revealed that C2 ceramide treatment on days 3-4 significantly induced *Sox1* mRNA with a peak on day 5 (Fig. 5J). Moreover, C2 ceramide treatment during days 3-5 also induced the mRNA expression of the neural differentiation markers *Sox1*, *Pax6*, and *Tubb3* (Supplementary Fig. S13). Thus, C2 ceramide treatment of EBs inhibits the expression of genes essential for PrS formation and, conversely, induces the expression of genes related to neurogenesis.

### Ceramide Alters Sphingolipid Metabolism and Glycerophospholipid Metabolism

**Figure 6.**
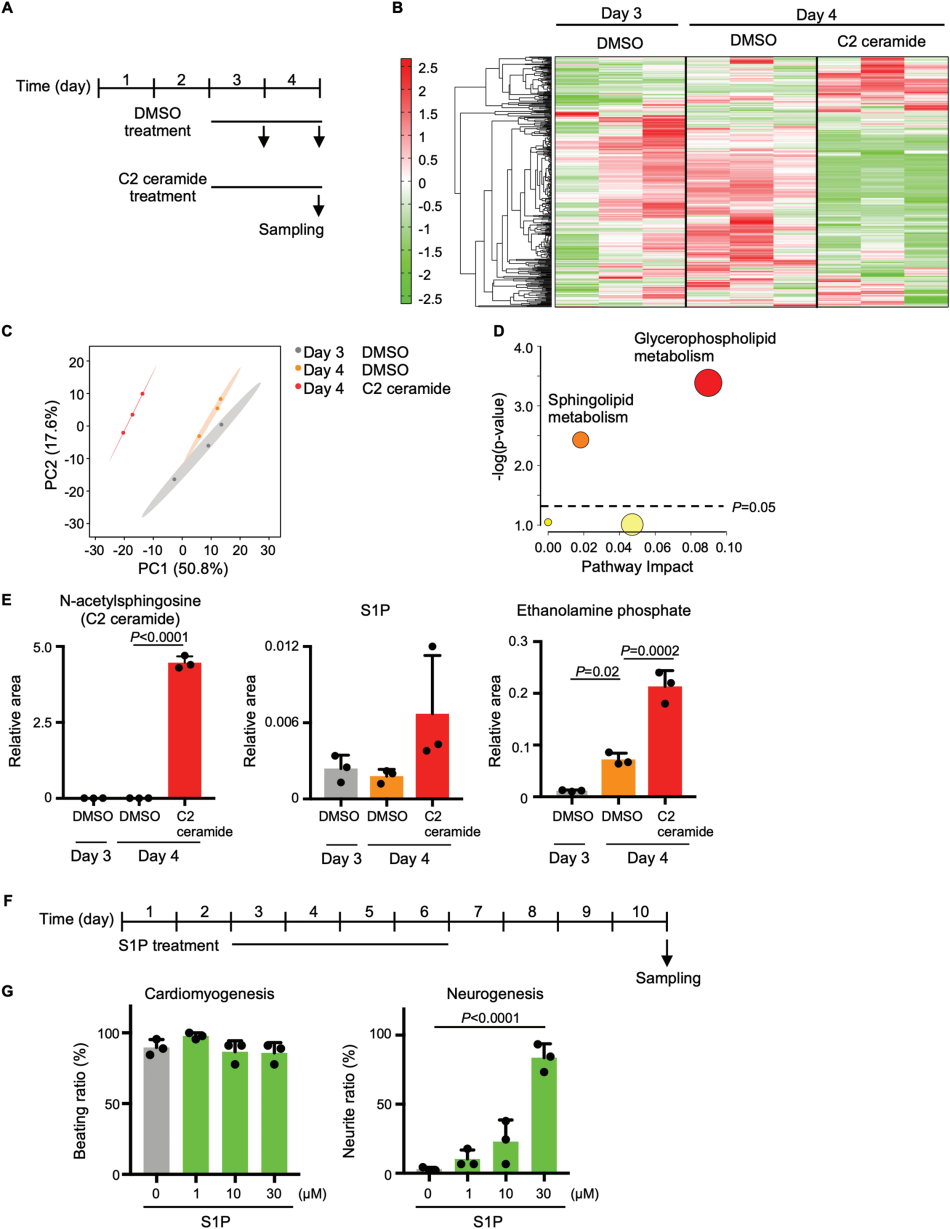
Effects of ceramide on cellular metabolism. (**A**) Experimental scheme for metabolomic analysis of C2 ceramide-treated EBs. (**B**) Clustering analysis of metabolites in EBs treated with either DMSO or 30 μM C2 ceramide for days 3-4. (**C**) PCA plot of metabolites in the DMSO- or C2 ceramide-treated EBs in (B). (**D**) Pathway analysis of metabolites in (B) whose levels were increased on day 4 compared to day 3 and altered by C2 ceramide treatment. (**E**) Quantitation of N-acetylsphingosine, S1P and ethanolamine phosphate in the EBs in (B). Data are the mean + s.d. of *n* = 3 biologically independent samples. Statistical analysis was performed by one-way ANOVA with the Tukey test. (**F**) Experimental scheme for the EB differentiation experiments in (G). (**G**) Quantitation of cardiomyogenesis and neurogenesis in EBs treated with the indicated concentrations of S1P for days 1-6. Data are the mean + s.d. of *n* = 3 biologically independent samples. Statistical analysis was performed by one-way ANOVA with the Tukey test.

To elucidate the metabolic changes that occur in cells during C2 ceramide treatment, a metabolomic analysis was conducted of EBs that were treated with DMSO or C2 ceramide from days 3-4, with metabolites extracted on day 3 or day 4 (Fig. 6A and Supplementary Table S12). Clustering and principal component analysis revealed that the metabolome was altered on day 4 in C2 ceramide-treated EBs compared to DMSO-treated EBs (Fig. 6B, C). Next, a pathway analysis was performed examining metabolites that were elevated from day 3 to day 4 of DMSO treatment and whose abundance changed on day 4 of C2 ceramide treatment compared to day 4 of DMSO treatment. The results showed that the sphingolipid and glycerophospholipid metabolic pathways were significantly altered (Fig. 6D). When metabolites of sphingolipid metabolism were examined individually, the expected significant increase in C2 ceramide (due to its addition as treatment) was readily detected (Fig. 6E). Sphingosine-1-phosphate (S1P), which is synthesized from ceramide, was present at equal concentrations on days 3 and 4 in DMSO-treated EBs, and tended to increase on day 4 in C2 ceramide-treated EBs. The amount of ethanolamine phosphate, a metabolite of S1P, rose significantly from day 3 to day 4 in DMSO-treated EBs, and was further increased on day 4 in C2 ceramide-treated EBs.

Because S1P also functions as a signaling molecule, we examined its role during the cardiac and neural differentiation of ES cells. We treated EBs with S1P on days 3-6 and examined its effects on cell beating and neurite formation on day 10 (Fig. 6F). Interestingly, S1P did not affect cell beating at the concentrations tested, but did significantly induce neurite formation in a dose-dependent manner (Fig. 6G). Since glycerophospholipid metabolism is coupled with sphingolipid metabolism and was markedly altered in C2 ceramide-treated EBs (Fig. 6D), we examined levels of 1-palmitoyl-glycero-3-phosphocholine, a type of lysophosphatidylcholine (LPC), as well as glycerophosphocholine, and found these to be increased on day 4 in DMSO-treated EBs (Supplementary Fig. S14A). On the other hand, the addition of C2 ceramide decreased the amount of these molecules detected on day 4. Levels of phosphocholine, a major metabolite in the choline metabolic pathway, were also decreased on day 4 of DMSO treatment and further decreased on day 4 in C2 ceramide-treated EBs. Since choline metabolism was altered by ceramide addition, phosphatidylcholine levels were measured on day 4 after day 3-4 treatment with C2 ceramide and were observed to be significantly reduced (Supplementary Fig. S14B). Taken together, these data indicate that C2 ceramide alters sphingolipid and glycerophospholipid metabolism, and that S1P induces neural differentiation.

### Inhibition of PrS Formation by Ceramide Promotes Neural Differentiation

**Figure 7.**
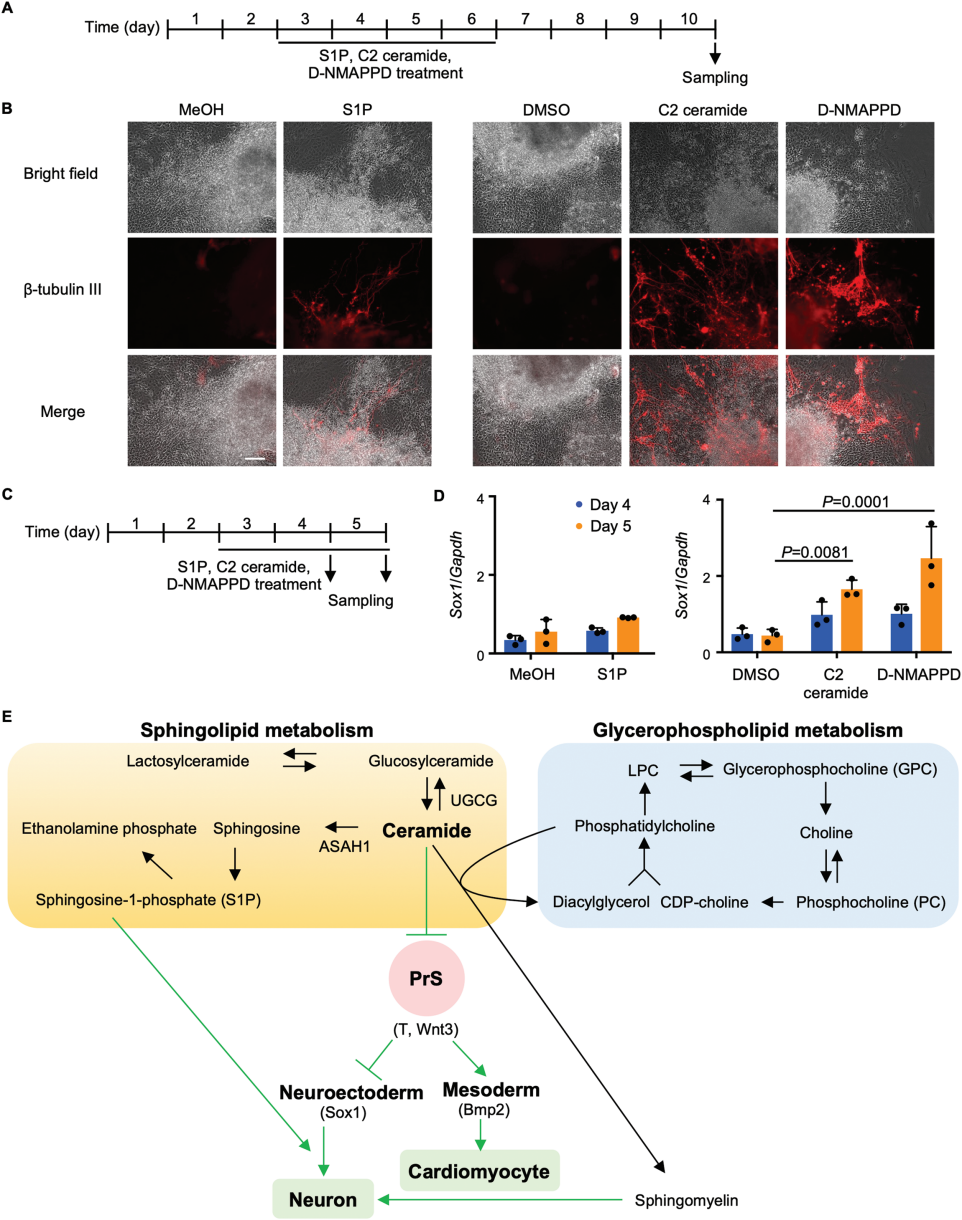
Effects of S1P, C2 ceramide, and D-NMAPPD on neurogenesis. (**A**) Experimental scheme for the EB drug treatments in (B). (**B**) Immunostaining with anti-β-tubulin III antibody to reveal morphological changes in EBs treated with methanol (MeOH), 30 μM S1P, DMSO, 30 μM C2 ceramide or 30 μM D-NMAPPD for days 3-6. Scale bar = 100 μm. Data are representative of 2 biologically independent experiments. (**C**) Experimental scheme for the EB drug treatments in (D). (**D**). Real-time PCR analysis of *Sox1* mRNA levels in EBs treated as in (C). Data are the mean + s.d. of *n* = 3 biologically independent samples. Statistical analysis was performed by one-way ANOVA with the Tukey test. (**E**) Scheme illustrating how ceramide metabolism might regulate cardiomyogenesis and neurogenesis in mouse EBs. Ceramide is involved in both sphingolipid (left) and glycerophosholipid (right) metabolism. High levels of ceramide induce neurogenesis at the expense of cardiomyogenesis mainly by inhibiting PrS formation. In addition, ceramide-derived S1P promotes the maturation of neuroectoderm to form neurons. Glycerophospholipid metabolism is altered during ceramide-induced neurogenesis.

Lastly, we compared the effects on EB neural differentiation of ASAH1 inhibition vs. treatment with S1P or C2 ceramide. As was shown in Fig. 2A, inhibition of ASAH1 by D-NMAPPD inhibits S1P synthesis from ceramide, resulting in ceramide accumulation. We treated EBs with either S1P (in methanol; MeOH), C2 ceramide (in DMSO), or D-NMAPPD (in DMSO) on days 3-6, and evaluated neurogenesis on day 10 (Fig. 7A). In control DMSO- or MeOH-treated EBs, almost no β-tubulin III-positive neurites were observed (Fig. 7B). In C2 ceramide- or D-NMAPPD-treated EBs, β-tubulin III-positive neurites were more numerous and formed in more regions than in S1P-treated EBs. To assess this difference at the gene expression level, we treated EBs with S1P, C2 ceramide, or D-NMAPPD on days 3-5, and collected samples on days 4 and 5 for real-time PCR analysis of *Sox1*, a marker of neural differentiation (Fig. 7C). No significant increase in *Sox1* mRNA was observed after S1P treatment on day 4 or 5 compared to the MeOH-treated control (Fig. 7D). In contrast, C2 ceramide or D-NMAPPD treatment significantly increased *Sox1* expression on day 5. These results suggest that the primary cause of ceramide-induced neural differentiation is the inhibition of PrS formation, and that S1P promotes the maturation of existing *Sox1*-positive neural progenitors (neuroectoderm). A model of our hypothesis is presented in Fig. 7E, with additional details presented in Supplementary Fig. S15.

## Discussion

PrS is a highly dynamic tissue, and its formation involves a wide range of cellular activities, including cell proliferation and migration, that lead to critical morphological changes.^33^ In previous work, we identified factors essential for PrS formation by screening a library of 1600 compounds with known targets and applying them to our in vitro EB differentiation system.^11^ We found that statins inhibit PrS formation because they block the mevalonate metabolic pathway, which leads to loss of the farnesylation of proteins such as nuclear lamin B1 and small G proteins.^11^ Similarly, we showed that the acetylcholine receptor inhibitor aprofene inhibits PrS formation by blocking the degradation of retinoic acid, thereby impeding the expression of PrS-related genes.^12^ We have also demonstrated that the p38 inhibitor SB203580 inhibits the spontaneous differentiation of ES cells into cardiomyocytes.^13^ These observations established that drug screening can pinpoint factors critical for PrS formation.

In the present study, we carried out KO mouse database screening to find genes involved in PrS formation that eluded identification in our chemical screens. Among these genes, many encode proteins functioning in glucose, mitochondrial, or nucleic acid metabolism, indicating that these pathways are essential for supplying the energy and substances necessary for PrS formation. Although we focused here on sphingolipid metabolism, we identified many other metabolic genes of potential future interest (Supplementary Figs. S1-S8), and expect that genes in some of the other 64 categories shown in Fig. 1B will also be important for PrS formation. Thus, the comprehensive screening results based on mouse lethal phenotypes presented in this study will be useful for further work aimed at understanding PrS formation.

Ceramide is a component of the lipid bilayer in a cell’s plasma membrane.^32^ In addition, ceramide functions intracellularly as a signaling molecule and is involved in cell differentiation, proliferation, and apoptosis.^34-36^ In this study, we have shown that ceramide also exerts reversible regulation of the expression of genes essential for PrS formation, such as *Brachyury T* and *Wnt3*. This reversibility may allow for flexible spatio-temporal control of PrS formation to ensure proper embryonic development in vivo under a range of microenvironmental conditions.

Our results suggest that concentrations of ceramide are low in the PrS region of a developing EB. The de novo ceramide synthesis pathway begins with L-serine and palmitoyl CoA (Fig. 2A), and L-serine is synthesized from intermediates of glycolysis. A decrease in glycolytic intermediates might result in decreased serine biosynthesis, leading to a decrease in local ceramide synthesis (Supplementary Fig. S1). Indeed, it has been reported that metabolic switching from glycolysis to oxidative phosphorylation, which generates more energy in the form of ATP, occurs during mesendoderm differentiation in human pluripotent stem cells.^37^

S1P is a lipid mediator that is derived from sphingolipids such as ceramide and is responsible for signaling through receptors on cell membranes.^38,39^ KO mice lacking sphingosine kinases, which produce S1P, show severe defects in neurogenesis, suggesting the involvement of S1P in neural structure formation, but the point of action of S1P was heretofore unknown.^40^ Our results suggest that S1P promotes neural maturation rather than the induction of neural progenitor cells.

It was previously reported that the ratio of the choline metabolites phosphocholine (PC) and glycerophosphocholine (GPC) (PC/GPC ratio) is decreased during PrS formation in mouse and human ES cells.^41,42^ However, the physiological meaning and the mechanism driving this change in the PC/GPC ratio were not resolved. We obtained similar results in our examination of PrS formation and neural differentiation (Supplementary Fig. S14C). Phosphatidylcholine reacts with ceramide to synthesize sphingomyelin, a component of the myelin sheath. During PrS formation, the consumption of phosphatidylcholine by this reaction is decreased, elevating the amount of phosphatidylcholine entering the choline metabolic cycle; this entrance increases the amount of GPC and decreases the PC/GPC ratio. On the other hand, when neural differentiation is promoted by ceramide, phosphatidylcholine is consumed for sphingomyelin synthesis, decreasing GPC and raising the PC/GPC ratio. Thus, changes in the PC/GPC ratio may reflect the opposing metabolic demands of PrS formation versus neural differentiation.

It is difficult to accurately evaluate ceramide concentrations within the tiny amount of tissue represented by the PrS. We measured ceramide levels in whole EBs after treatment with the UGCG inhibitor NB-DNJ. The increase in the level of a specific species of ceramide (C16:0), and the alterations to the levels of various ceramide-related metabolites (sphingomyelin, galactosyl/glucosylceramide and lactosylceramide), indicate that UGCG is a physiological regulator of ceramide levels (Supplementary Fig. S11). ASAH1 is also involved, since previous studies have shown that ceramide accumulates in various tissues upon ASAH1 inhibition. For example, in mice heterozygous for an *Asah1* mutation, ceramide accumulates in the liver.^43^ In humans, patients with Farber disease show reduced activity of ASAH1 due to their genetic defects and exhibit ceramide accumulation in the kidney.^44^ Taken together, these results strongly indicate that UGCG and ASAH1 are important for regulating ceramide levels.

Several groups have reported that PrS formation inhibits neural differentiation. For example, the FGF/ERK pathway^45^ and Tbx6^46^ are necessary for PrS formation, and the inhibition of these pathways induces neural differentiation. Interestingly, our RNA-seq analysis found that mRNA levels of *Fgf8* and *Tbx6* were both decreased by C2 ceramide treatment (Supplementary Fig. S12C). Thus, ceramide may be an upstream regulator of the Fgf8 and Tbx6 pathways involved in PrS formation.

We note here that our mouse results will likely be relevant to human embryogenesis. We have previously demonstrated the usefulness of our mouse in vitro ES cell differentiation system as a tool that can recapitulate in vivo events in humans. For example, statins are contraindicated in pregnant women because of their potential teratogenic effects.^47^ We used our system to show that administration of statins to mouse and zebrafish embryos caused abnormal embryonic development in both cases.^11^ These findings established that our mouse ES cell system can reliably replace animal experiments for assessing drug toxicity, thereby not only providing insights into consequences for humans but also improving animal welfare. Our present study has further established that our system is useful for elucidating general molecular mechanisms of PrS formation in mammals.

## Conclusion

Using our experimental system in which epiblast differentiates into PrS, mesendoderm, and then cardiomyocytes under normal conditions, we have shown that exogenous S1P does not affect PrS formation but does induce neural maturation of *Sox1*-positive neuroectoderm. Furthermore, we have established that ceramide accumulation inhibits PrS formation and instead induces neuroectoderm formation, resulting in the appearance of β-tubulin III-positive neural cells (Supplementary Fig. S15). These data collectively indicate that PrS formation inhibits neural differentiation through control of ceramide levels. However, the underlying molecular mechanisms operating during this regulation remain unclear. Future work is needed to clarify how these fundamental processes are linked.

## Supplementary Material

sxad071_suppl_Supplementary_Methods

sxad071_suppl_Supplementary_Tables_S1-S12

sxad071_suppl_Supplementary_Figures_S1-S15

## Data Availability

RNA-sequencing data have been deposited in the GEO under the accession code GEO207850. All other data and materials used in this study are available from the corresponding authors upon reasonable request.
