# Supplementary material for: Lethal Phenotype-Based Database Screening Identifies Ceramide as a Negative Regulator of Primitive Streak Formation
Source: Stem Cells. 2023 Oct 11;41(12):1142–56. doi: 10.1093/stmcls/sxad071 (PMC10722545; doi:10.1093/stmcls/sxad071)
Supplement: sxad071_suppl_Supplementary_Methods [file sxad071_suppl_supplementary_methods.docx]

**SUPPLEMENTARY METHODS for manuscript #SC-22-0123.R1**

**Reagents and antibodies**

Reagents used in this study were as follows: myriocin (M1177; Sigma, Burlington, USA), N-butyldeoxynojirimycin (NB-DNJ) (B8299; Sigma, Burlington, USA), sphingosine 1-phosphate (S9666; Sigma-Aldrich, Burlington, USA), N-[(1R,2R)-2-hydroxy-1-(hydroxymethyl)-2-(4-nitrophenyl) ethyl]-tetradecanamide (D-NMAPPD) (10006305; Cayman, Michigan, USA), C2 ceramide (860502P; Avanti Polar Lipids, Alabama, USA), and C16 ceramide (860516P; Avanti Polar Lipids, Alabama, USA). Anti-β-tubulin III monoclonal antibody (Tuj-1) (801201, BioLegend, San Diego, USA) and Alexa Fluor 546 goat anti-mouse secondary antibody (A11030, Invitrogen, Massachusetts, USA) were used for neurite detection.

**Whole mount *in situ* hybridization**

Whole mount *in situ* hybridization was performed as previously described^11,48^. Briefly, EBs were fixed overnight at 4°C in 4% PFA/DEPC-PBS, and dehydrated and rehydrated through a methanol series. EBs were digested in 10 μg/ml proteinase K at room temperature for 4 min, followed by the addition of 2 mg/ml glycine PBST solution to stop the reaction. After re-fixation in 1 ml 4% PFA/0.2% glutaraldehyde/PBST, EBs were prehybridized to block non-specific RNA-probe interactions. EBs were then incubated with specific digoxigenin (DIG)-labelled RNA probes overnight at 65°C. DIG-labelled RNA probes were detected by incubation with a 1:500 dilution of anti-DIG antibody conjugated to alkaline phosphatase (AP) (11093274910; Roche, Basel, Switzerland), followed by staining with BM Purple AP Substrate (11442074001; Roche, Basel, Switzerland). The following primer set was used: *Brachyury T*, 5’-TTTGAATTCC AGTTAATCAGAGTCCTTTG-3’ and 5’-TTTAAGCTTACCAGGTGCTA TATATTGCC-3’.

**Metabolomic analysis**

Metabolomic analysis was entrusted to Human Metabolome Technologies, Inc. (HMT, Yamagata, Japan). To inactivate enzymes prior to LC-TOF-MS analysis, EBs were washed with 10 ml 5% mannitol solution and treated with 1 ml ethanol containing internal standards. Samples were homogenized by ultra-sonication for 5 min on ice, followed by centrifugation at 4,400 ×g at 4°C for 5 min. Supernatants were dried and dissolved in 200 μl 50% 2-propanol. LC-TOF-MS was carried out using an Agilent 1200 series RRLC system SL. Compounds were analyzed in both positive and negative ion polarity mode. Detected peaks were processed using Master Hands ver. 2.17.1.11. The “relative area” was calculated as the area of the peak obtained by mass spectrometry corrected by the value of the internal standard metabolites and the total protein content of the cells in the sample. The HMT metabolite library was used to annotate the peaks based on their m/z values and retention time.

**RNA sequencing and analysis**

Total RNA was extracted by using RNeasy Mini Kits (74104; QIAGEN, Hilden, German) according to the manufacturer’s instructions. Extracted RNA was incubated with DNase I (2270B; Takara, Shiga, Japan) to degrade the genome. RNA-sequencing analysis was entrusted to Takara Bio Inc. (Shiga, Japan). SMART-Seq v4 Ultra Low Input RNA Kit for Sequencing (Clontech), Nextera XT DNA Library Prep Kit (Illumina), Nextera XT Index Kit v2 (Illumina) were used to amplify double-stranded cDNAs and prepare the sequencing library. RNA-seq was performed using NovaSeq 6000 plus NovaSeq Control Software v1.6.0, Real Time Analysis (RTA) v3.4.4, and Bcl2fastq2 v2.20. Sequence data were analyzed using DRAGEN Bio-IT Platform v3.6.3 (Illumina) with GRCm38 Release m25 as the reference sequence. Results are expressed in transcripts per million (TPM) rather than FPKM values because TPM better reflects gene expression levels and is now frequently used as a normalization method^49^. The RNA-seq data have been deposited into Gene Expression Omnibus (GEO) and are available under the number GSE207850.

For gene expression analysis, the logarithm of the TPM value (bottom 2) was calculated and genes whose expression showed a change greater than 2-fold or less than 0.5-fold were selected. Absolute values of fold change were calculated and data with fold change greater than a threshold value were extracted. Functional pathway and clustering analyses were performed using the list, and Gene Ontology analysis was performed by entering Entrez Gene IDs into the Database for Annotation, Visualization and Integrated Discovery (DAVID) (https://david.ncifcrf.gov/) query.

**Real-time PCR analysis**

Real-time PCR analysis was performed as previously described^11,12,50^. Briefly, EBs were collected and immediately suspended in TRIzol Reagent (15596018; Thermo Fisher Scientific, Massachusetts, USA). RNA was extracted according to the manufacturer’s instructions. Total RNA (4 μg) was used to synthesize cDNAs at 42°C for 90 min in a 40 μl reaction containing 2.5 μM oligo-d(T) primers, 0.125 mM of each deoxynucleoside triphosphate (dNTP), 16 U RNaseOUT (10777019; Invitrogen, Massachusetts, USA), and 80 U Superscript III RNase H reverse transcriptase (18080044; Invitrogen, Massachusetts, USA) according to the manufacturer’s instructions. Quantitative real-time RT-PCR reactions were performed using the CFX96 real-time system (Bio-Rad, California, USA). Primer sequences are listed in Supplementary Table S12.

**Lipid extraction and phosphatidylcholine measurement**

Lipid extraction was performed as described previously^51^. Briefly, EBs were washed twice with 1 ml PBS and homogenized in 200 μl 1:2 (v/v) chloroform:methanol solution. CHCl_3_ and H_2_O (1.25 ml each) were added to the homogenate followed by vigorous vortexing. Samples were centrifuged at 1,000 rpm at room temperature for 5 min to yield a two-phase system. The organic bottom phase was recovered and washed with 5:5:4 (v/v/v) chloroform:methanol:1M NaCl solution, followed by centrifugation at 1,000 rpm at room temperature for 5 min. The organic bottom phase was recovered and dried under a gentle stream of nitrogen. Phosphatidylcholine analysis was performed using a phosphatidylcholine colorimetric/fluorometric assay kit (K576; BioVision) according to the manufacturer’s protocol. Measurements were performed at OD570 nm using a Multiskans microplate spectrophotometer (Thermo Scientific).

**Ceramide measurement**

Ceramide measurement was entrusted to Lipidome Lab Co. (Akita, Japan). EBs were homogenized in methanol to create a mixture with a protein concentration of 10 μg/μL. Total lipids were extracted from 20 μL of this protein mixture using the Bligh & Dyer method^51^. The acidic phospholipid fraction was purified from the total lipid fraction using an anion exchange column. Acidic phospholipids were measured using ACQUITY UPLC H-Class and Xevo TQ-XS instruments (Waters), with analysis performed by MassLynx4.2 (Waters). Ceramide levels were calculated as the area of the peak obtained by mass spectrometry corrected by the value of the internal standard metabolites and the protein concentration.

**Supplementary References**

48. Piette, D., Hendrickx, M., Willems, E., Kemp, C. R. & Leyns, L. An optimized procedure for whole-mount in situ hybridization on mouse embryos and embryoid bodies. *Nat. Protoc.* **3**, 1194–1201 (2008).

49. Wagner, G. P., Kin, K. & Lynch, V. J. Measurement of mRNA abundance using RNA-seq data: RPKM measure is inconsistent among samples. *Theory Biosci.* **131**, 281–285 (2012).

50. Yu, R. *et al.* A Modified Murine Embryonic Stem Cell Test for Evaluating the Teratogenic Effects of Drugs on Early Embryogenesis. *PLoS One* **10**, e0145286 (2015).

51. Bligh, E.G. and Dyer, W. J. A rapid method of total lipid extraction and purification. *Can. J. Biochem. Physiol.* **37**, 911-917 (1959).
