# Supplementary material for: Lethal Phenotype-Based Database Screening Identifies Ceramide as a Negative Regulator of Primitive Streak Formation
Source: Stem Cells. 2023 Oct 11;41(12):1142–56. doi: 10.1093/stmcls/sxad071 (PMC10722545; doi:10.1093/stmcls/sxad071)
Supplement: sxad071_suppl_Supplementary_Figures_S1-S15 [file sxad071_suppl_supplementary_figures_s1-s15.pdf]

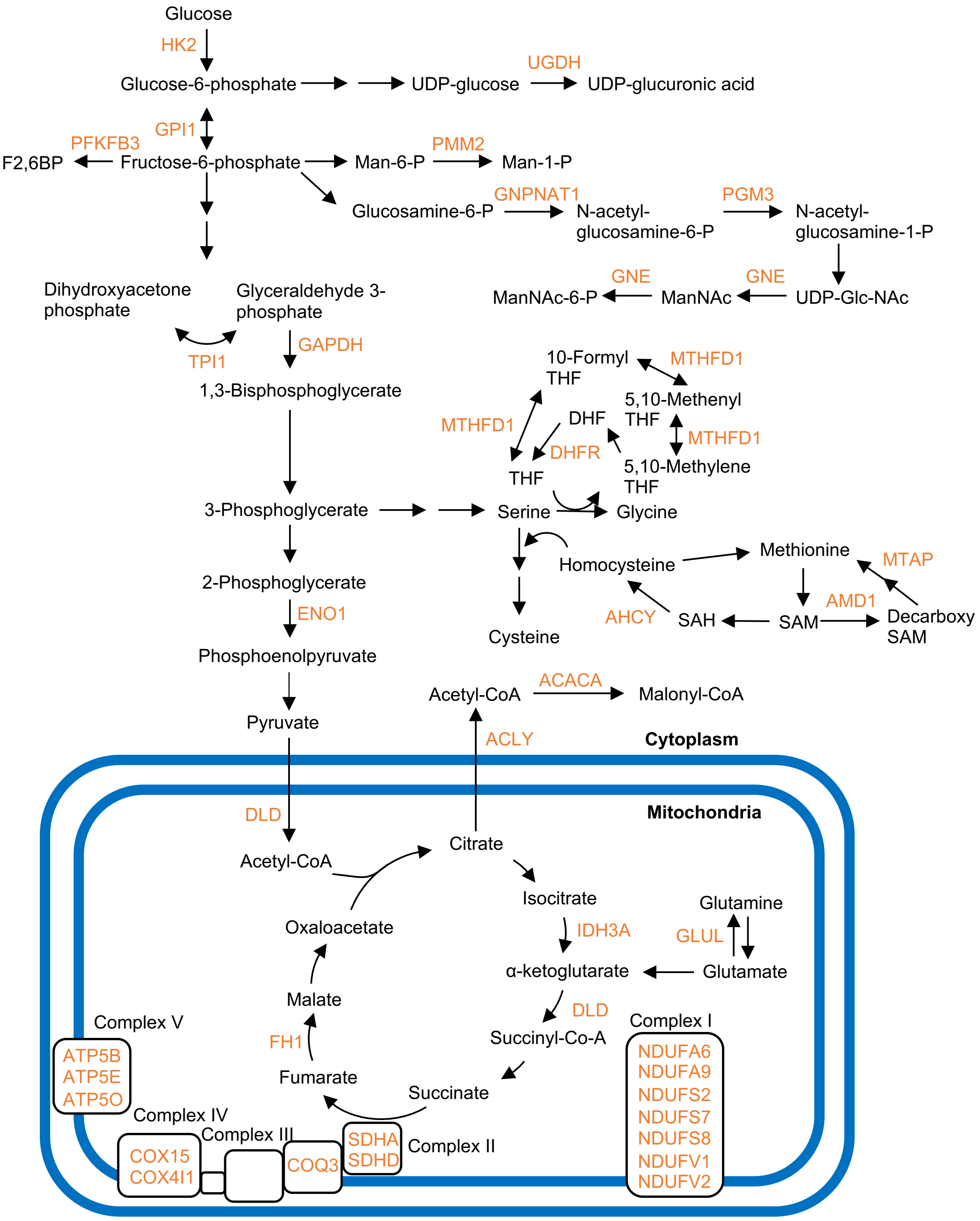

Supplementary Figure 1 Energy metabolism pathways
Enzymes identified in the database screening are shown in orange.

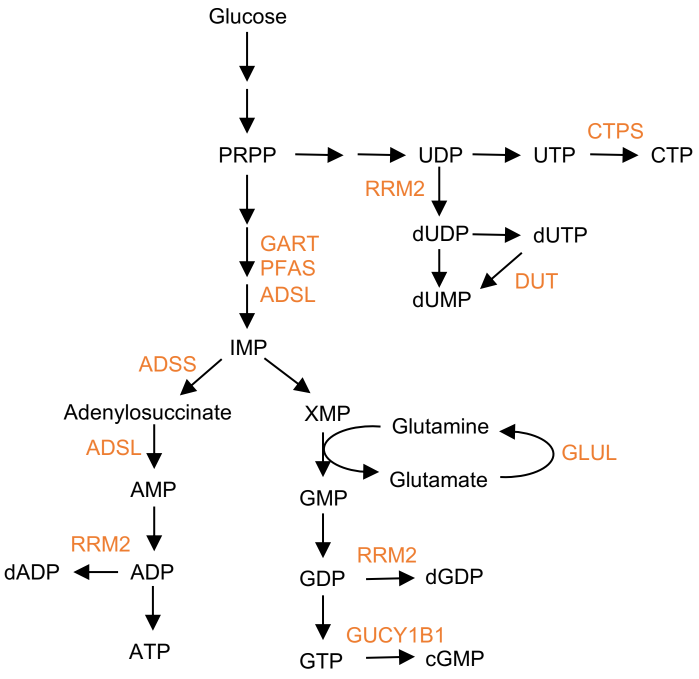

Supplementary Figure 2 Nucleotide metabolism pathways
Enzymes identified in the database screening are shown in orange.

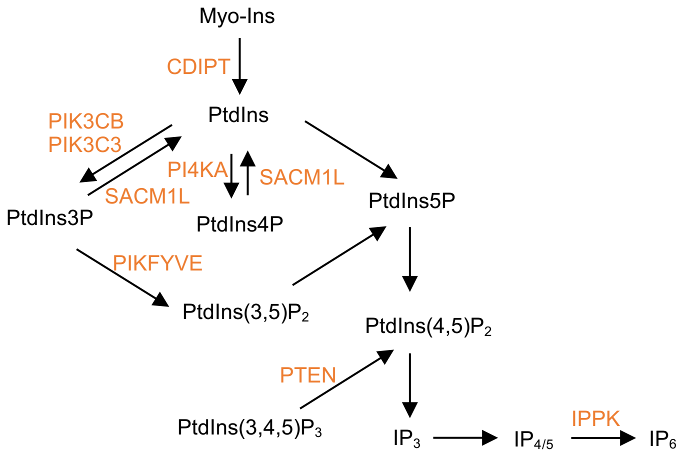

Supplementary Figure 3 Phosphoinositide metabolism pathways
Enzymes identified in the database screening are shown in orange.

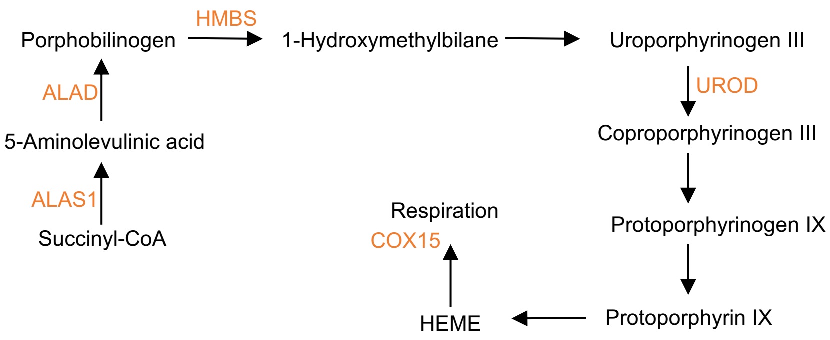

Supplementary Figure 4 Porphyrin and chlorophyll metabolism pathways
Enzymes identified in the database screening are shown in orange.

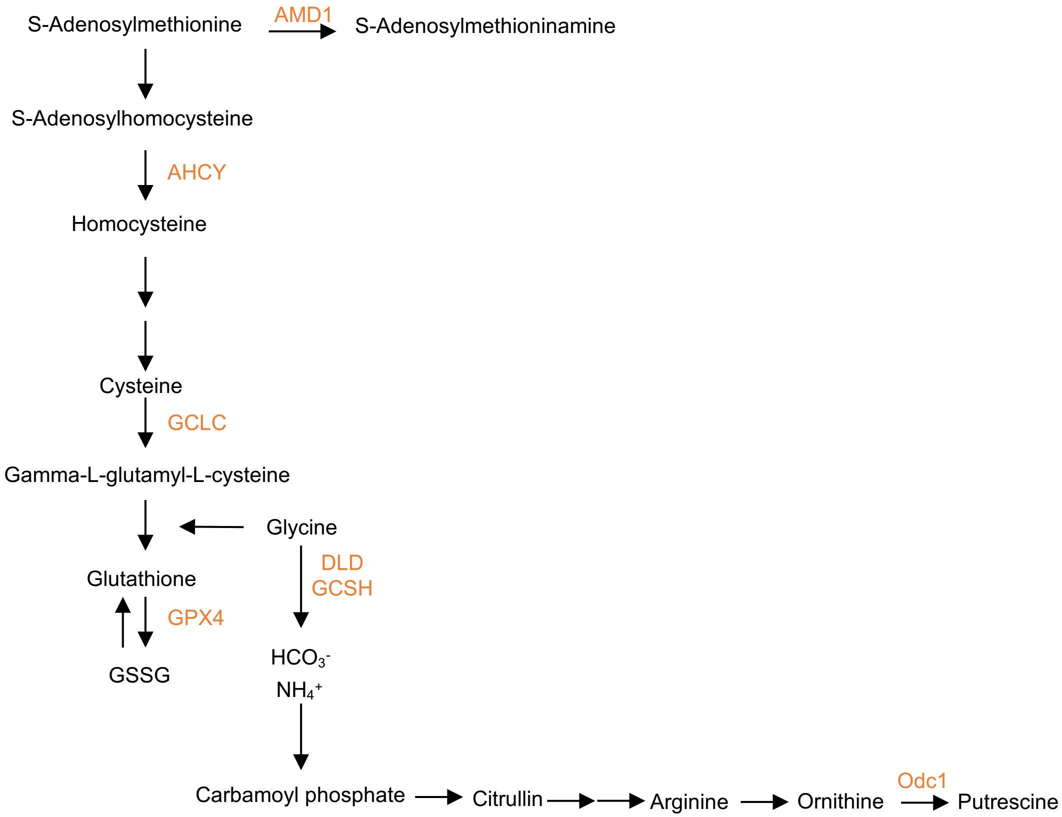

Supplementary Figure 5 Glutathione metabolism pathways
Enzymes identified in the database screening are shown in orange.

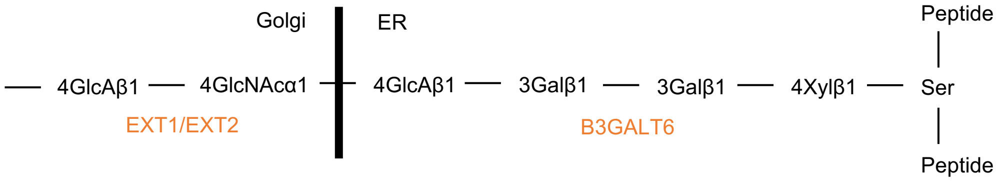

Supplementary Figure 6 Heparan sulfate biosynthesis pathways
Enzymes identified in the database screening are shown in orange.

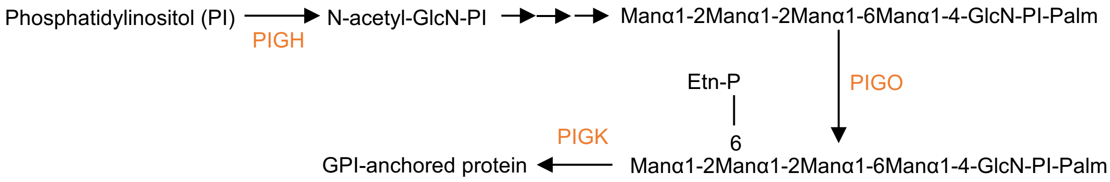

Supplementary Figure 7 Glycosylphosphatidylinositol(GPI)-anchor biosynthesis
Enzymes identified in the database screening are shown in orange.

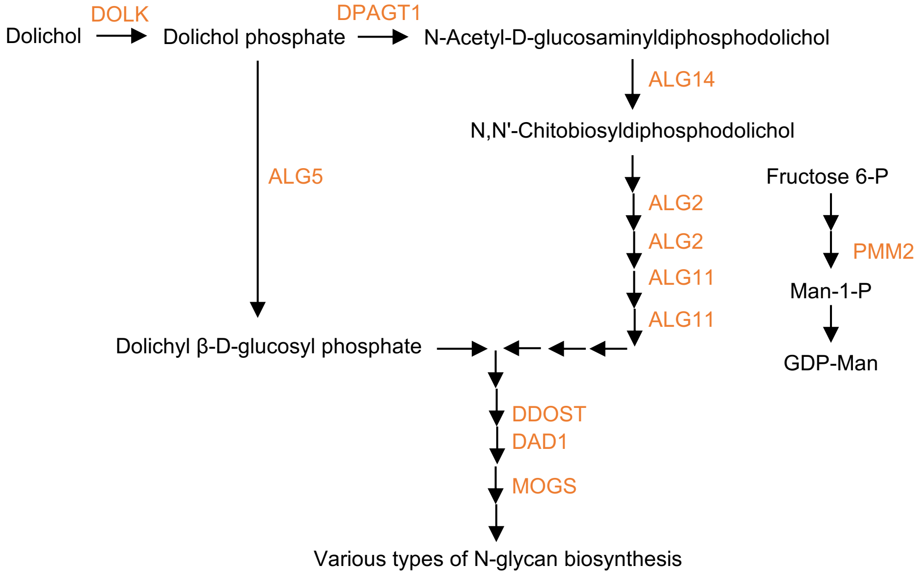

Supplementary Figure 8 N-glycan biosynthesis pathways
Enzymes identified in the database screening are shown in orange.

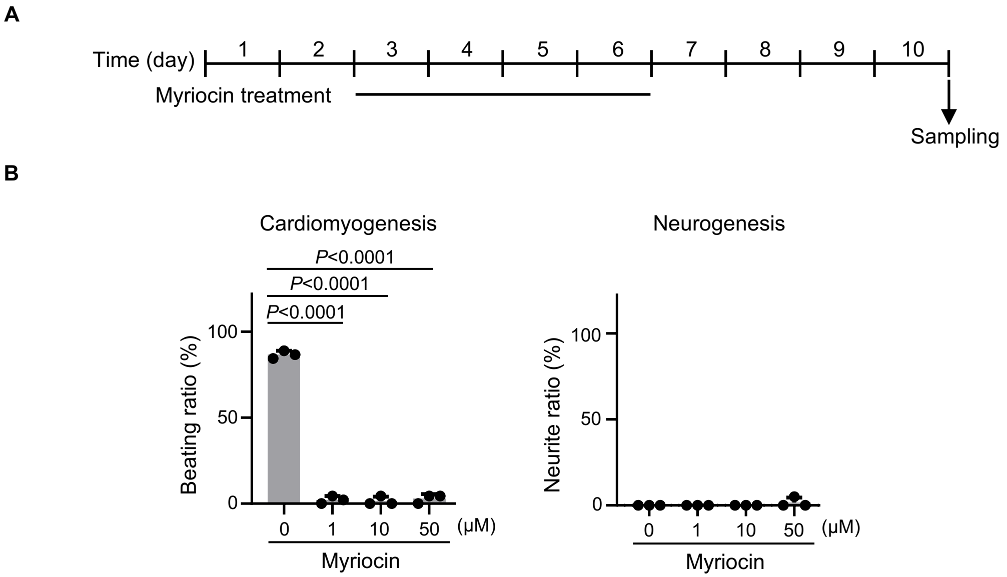

Supplementary Figure 9 Effects of Myriocin on EB differentiation.
(A) Experimental scheme. (**B)** Dose-dependent effects of Myriocin on cardiomyogenesis and neurogenesis in EBs treated with the indicated concentrations of myriocin for days 3-6. Data are the mean+s.d. *n*=3 biologically independent samples. Statistical analysis was performed by one-way ANOVA with the Tukey test.

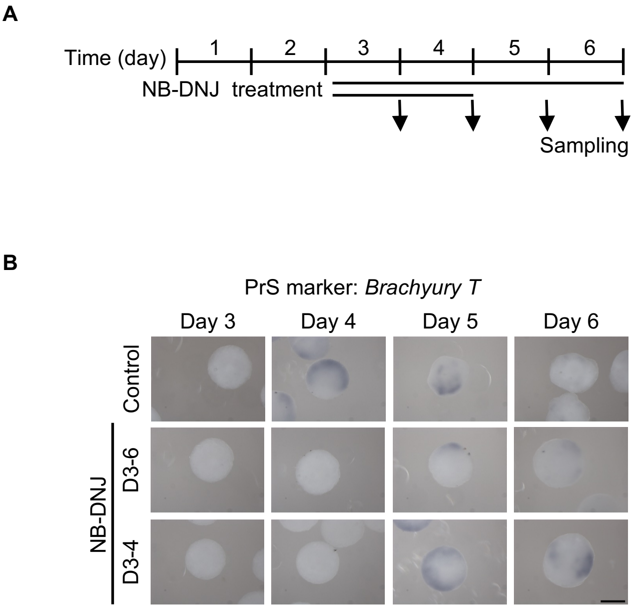

Supplementary Figure 10 *Brachyury T* expression pattern during NB-DNJ treatment.
(**A**) Experimental scheme for the *in situ* experiments in B. (**B**) Microscopic images of *in situ* hybridization to detect *Brachyury T* on the indicated days in EBs treated with water (control) or 50 μM NB-DNJ for days 3-4 or days 3-6 of *in vitro* differentiation. Scale bar, 100 μm. Data are representative of two biologically independent experiments.

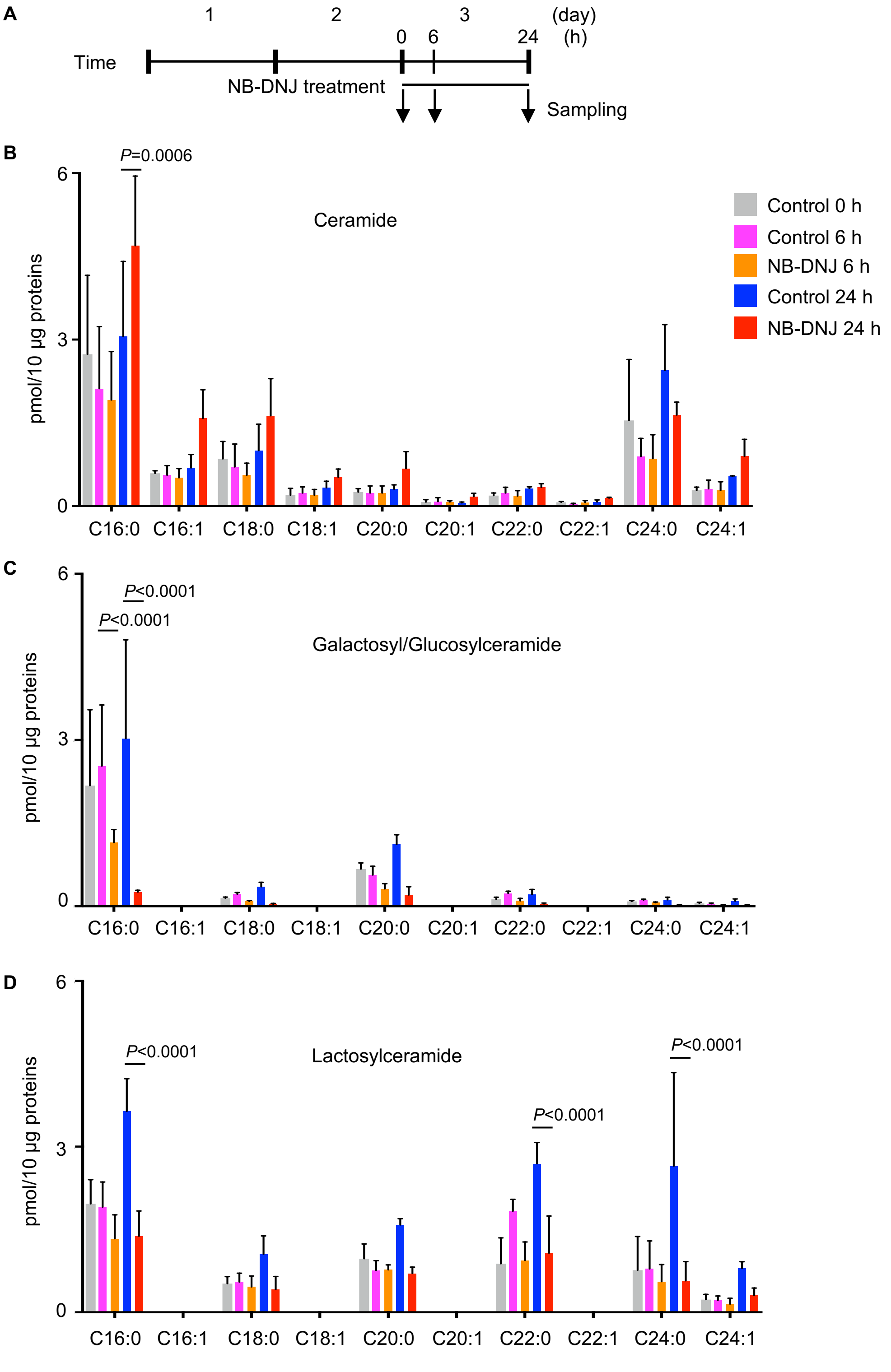

Supplementary Figure 11 Effects of NB-DNJ on levels of ceramide and ceramide-related metabolites.
(**A**) Experimental scheme for ceramide measurement in B, C, D. The amounts of (**B**) Ceramide, (**C**) Galactosyl/Glucosylceramide, (**D**) Lactosylceramide in EBs treated with DMSO, 50 μM NB-DNJ at the indicated times. Data are the mean+s.d. *n*=3 biologically independent samples. Statistical analysis was performed by two-way ANOVA with the Tukey test.

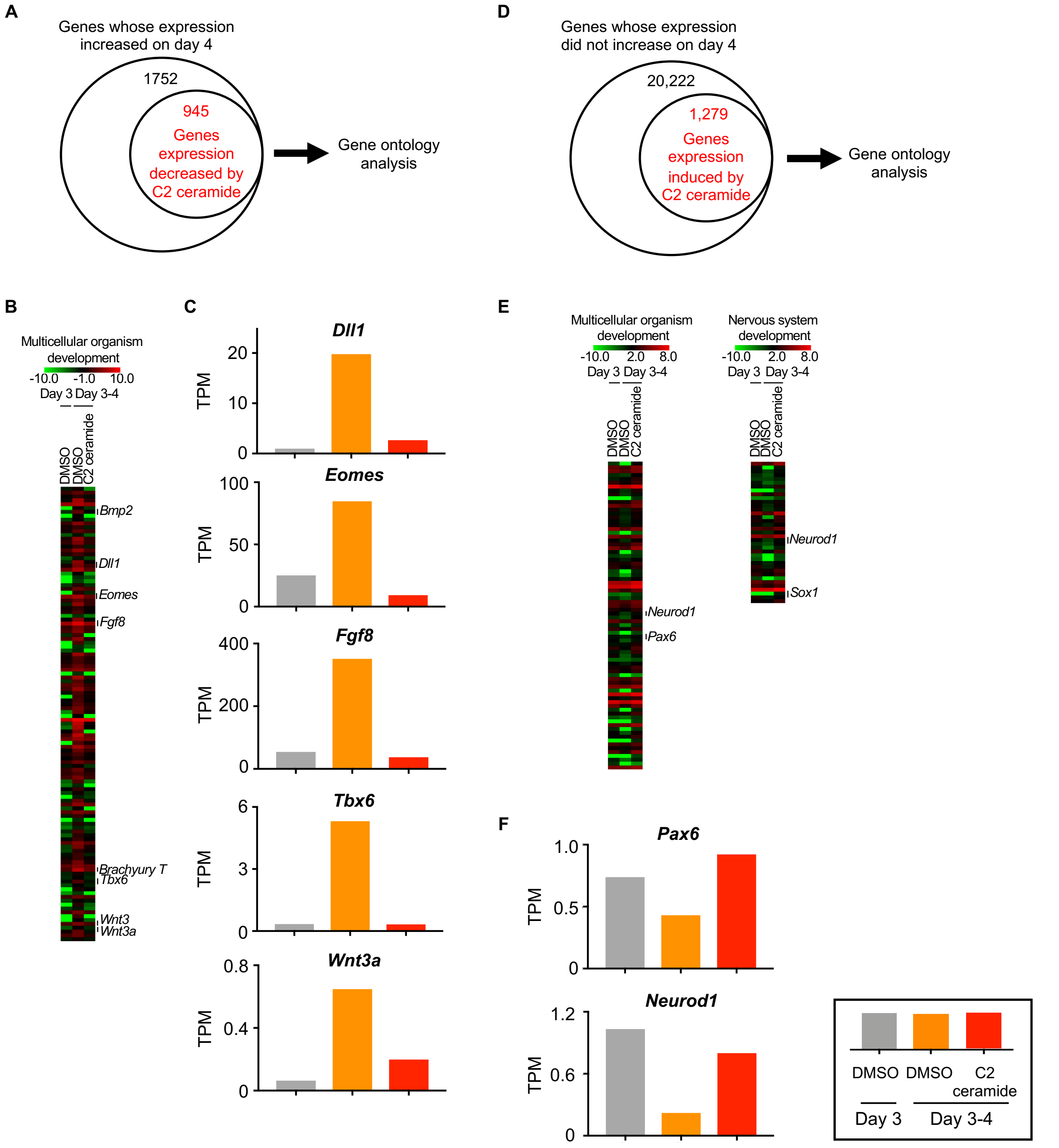

Supplementary Figure 12 Effects of ceramide on the transcriptome of mouse EBs.
(**A**) Scheme of GO analysis of genes whose expression levels increased during PrS formation but were inhibited by 30 μM C2 ceramide treatment. (**B**) Expression levels of genes related to "Multicellular organism development". Genes, which are reported to be involved in primitive streak formation, are indicated. (**C**) Levels of *Dll1*, *Eomes*, *Fgf8*, *Tbx6*, and *Wnt3a* mRNAs in EBs treated with DMSO (vehicle control) or 30 μM C2 ceramide for days 3-4 as determined by RNA-sequencing analysis. (**D**) Scheme of GO analysis of genes whose expression did not increase during PrS formation, but were induced by 30 μM C2 ceramide treatment. (**E**) Expression levels of genes related to "Multicellular organism development" and "Nervous system development". *Neurod1*, *Pax6* and *Sox1* are indicated. (**F**) Levels of *Pax6* and *Neurod1* mRNAs in EBs treated with DMSO (vehicle control) or 30 μM C2 ceramide for days 3-4 as determined by RNA-sequencing analysis.

A

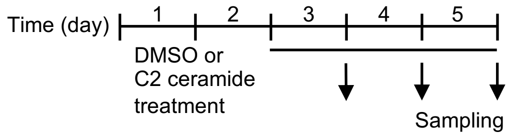

B

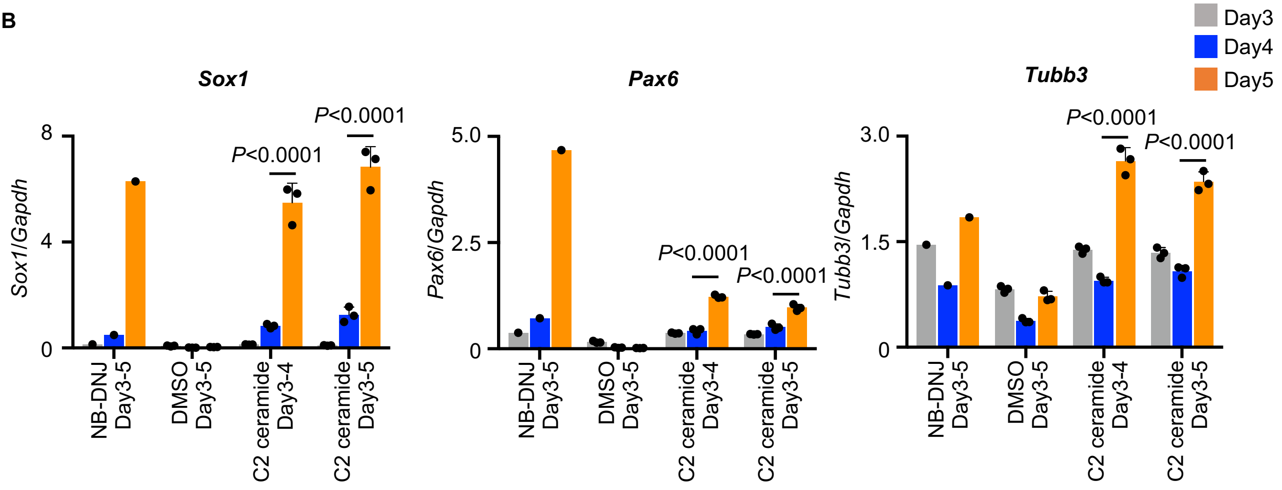

Supplementary Figure 13 Effects of ceramide on expression of genes for neurogenesis.
(**A**) Experimental scheme for the real-time PCR experiments in B. (**B**) Real-time PCR analysis to see the effect of C2 ceramide on the expression of *Sox1*, *Pax6* and *Tubb3*. EBs were treated with 45 μM C2 ceramide for different time duration. *n*=3 biologically independent samples. Data are shown mean+s.d. Statistical analysis was performed by two-way ANOVA with the Tukey test.

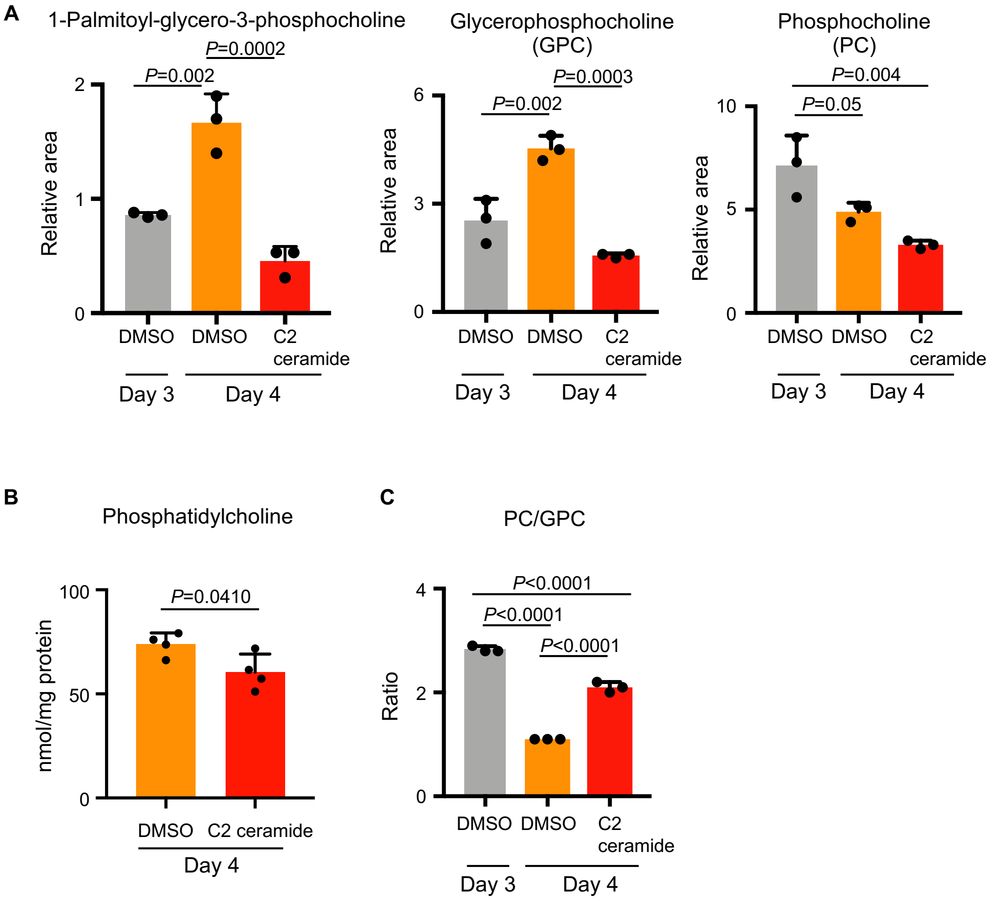

Supplementary Figure 14 Effects of C2 ceramide on glycerophospholipid metabolism of mouse EBs
(**A**) Quantification of 1-palmitoyl-glycero-3-phosphocholine, glycerophosphocholine, and phosphocholine in EBs treated on days 3-4 with 30 μM C2 ceramide. Data are the mean+s.d. *n*=3 biologically independent samples. Statistical analysis was performed by one-way ANOVA with the Tukey test. (**B**) Absolute amount of phosphatidylcholine in EBs on day 4 after the treatment with 30 μM C2 ceramide. Data are the mean+s.d. *n*=4 biologically independent samples. Statistical analysis was performed by two-tailed unpaired *t* test. (**C**) The ratio of phosphocholine/glycerophosphocholine in EBs treated on days 3-4 with 30 μM C2 ceramide. Data are the mean+s.d. *n*=3 biologically independent samples. Statistical analysis was performed by one-way ANOVA with the Tukey test.

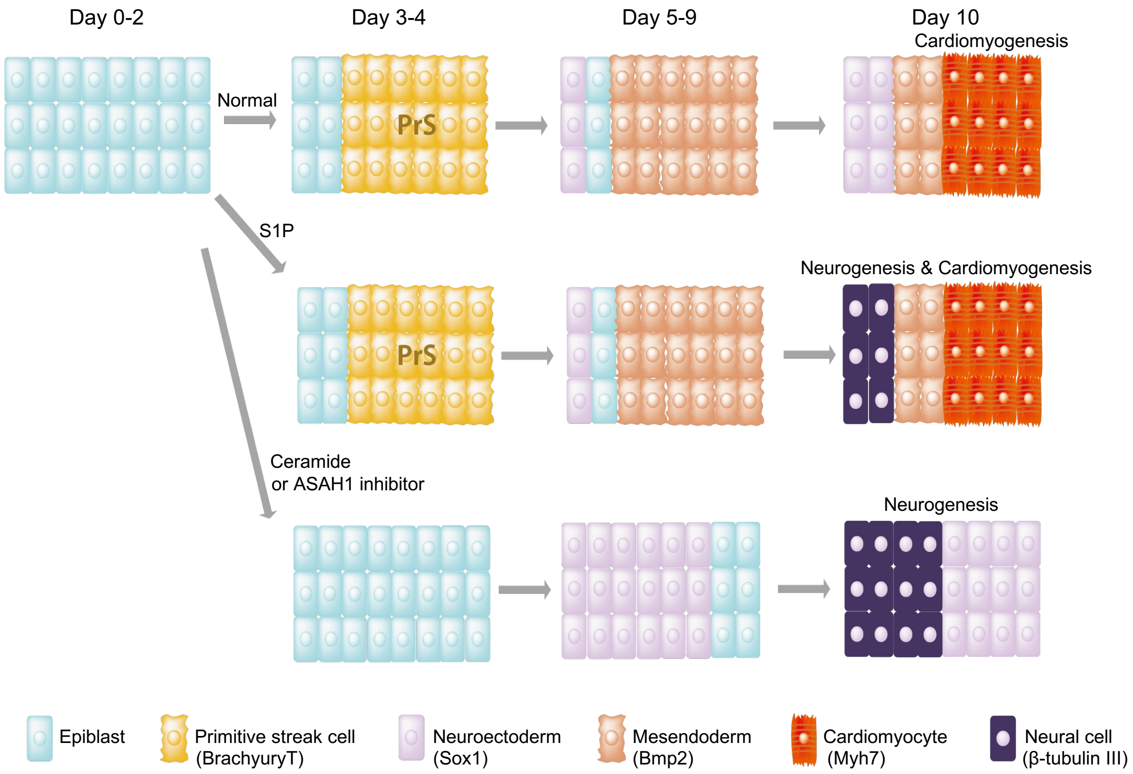

Supplementary Figure 15 Effects of S1P, ceramide, or an ASAH1 inhibitor on epiblast differentiation
Scheme illustrating how epiblasts differentiate into PrS cells and then cardiomyocytes and neural cells over time, and how this flow is affected by S1P, ceramide or ASAH1 inhibitor. Cardiomyogenesis and neurogenesis normally arise in spatially different parts of the embryo. Under normal conditions, PrS formation leads to cardiomyogenesis. S1P treatment induces the neurogenesis of Sox1-positive cells without affecting cardiomyogenesis. However, ceramide treatment inhibits PrS formation, blocks cardiomyogenesis and induces neurogenesis.
